# Comparison of weather station and climate reanalysis data for modelling temperature-related mortality

**DOI:** 10.1038/s41598-022-09049-4

**Published:** 2022-03-25

**Authors:** Malcolm N. Mistry, Rochelle Schneider, Pierre Masselot, Dominic Royé, Ben Armstrong, Jan Kyselý, Hans Orru, Francesco Sera, Shilu Tong, Éric Lavigne, Aleš Urban, Joana Madureira, David García-León, Dolores Ibarreta, Juan-Carlos Ciscar, Luc Feyen, Evan de Schrijver, Micheline de Sousa Zanotti Stagliorio Coelho, Mathilde Pascal, Aurelio Tobias, Multi-Country Multi-City (MCC) Collaborative Research Network, Barrak Alahmad, Rosana Abrutzky, Paulo Hilario Nascimento Saldiva, Patricia Matus Correa, Nicolás Valdés Orteg, Haidong Kan, Samuel Osorio, Ene Indermitte, Jouni J. K. Jaakkola, Niilo Ryti, Alexandra Schneider, Veronika Huber, Klea Katsouyanni, Antonis Analitis, Alireza Entezari, Fatemeh Mayvaneh, Paola Michelozzi, Francesca de’Donato, Masahiro Hashizume, Yoonhee Kim, Magali Hurtado Diaz, César De la Cruz Valencia, Ala Overcenco, Danny Houthuijs, Caroline Ameling, Shilpa Rao, Xerxes Seposo, Baltazar Nunes, Iulian-Horia Holobaca, Ho Kim, Whanhee Lee, Carmen Íñiguez, Bertil Forsberg, Christofer Åström, Martina S. Ragettli, Yue-Liang Leon Guo, Bing-Yu Chen, Valentina Colistro, Antonella Zanobetti, Joel Schwartz, Tran Ngoc Dang, Do Van Dung, Yuming Guo, Ana M. Vicedo-Cabrera, Antonio Gasparrini

**Affiliations:** 1 Department of Public Health, Environments and Society, London School of Hygiene & Tropical Medicine, London, UK; 2 Department of Economics, Ca’ Foscari University of Venice, Venice, Italy; 3 The Centre on Climate Change & Planetary Health, London School of Hygiene & Tropical Medicine, London, UK; 4 Forecast Department, European Centre for Medium-Range Weather Forecast (ECMWF), Reading, UK; 5 Ф-Lab, European Space Agency (ESA-ESRIN), Frascati, Italy; 6 Department of Geography, University of Santiago de Compostela, Santiago de Compostela, Spain; 7 CIBER de Epidemiología y Salud Pública (CIBERESP), Madrid, Spain; 8 Institute of Atmospheric Physics of the Czech Academy of Sciences, Prague, Czech Republic; 9 Faculty of Environmental Sciences, Czech University of Life Sciences, Prague, Czech Republic; 10 Department of Family Medicine and Public Health, University of Tartu, Tartu, Estonia; 11 Department of Statistics, Computer Science and Applications ‘G. Parenti’, University of Florence, Florence, Italy; 12 Shanghai Children’s Medical Center, Shanghai Jiao Tong University School of Medicine, Shanghai, China; 13 School of Public Health, Institute of Environment and Population Health, Anhui Medical University, Hefei, China; 14 School of Public Health and Social Work, Queensland University of Technology, Brisbane, QLD Australia; 15 Center for Global Health, School of Public Health, Nanjing Medical University, Nanjing, China; 16 Air Health Science Division, Health Canada, Ottawa, ON Canada; 17 School of Epidemiology and Public Health, University of Ottawa, Ottawa, ON Canada; 18 Department of Environmental Health, Instituto Nacional de Saúde Dr Ricardo Jorge, Porto, Portugal; 19 EPIUnit—Instituto de Saúde Pública, Universidade do Porto, Porto, Portugal; 20 The Joint Research Center (JRC), European Commission, Seville, Spain; 21 The Joint Research Center (JRC), European Commission, Ispra, Italy; 22 Graduate School of Health Science, University of Bern, Bern, Switzerland; 23 Institute of Social and Preventive Medicine, University of Bern, Bern, Switzerland; 24 Oeschger Center for Climate Change Research, University of Bern, Bern, Switzerland; 25 Faculty of Medicine, University of São Paulo, São Paulo, Brazil; 26 Santé Publique France, Department of Environmental and Occupational Health, French National Public Health Agency, Saint Maurice, France; 27 Institute of Environmental Assessment and Water Research (IDAEA), Spanish Council for Scientific Research (CSIC), Barcelona, Spain; 28 School of Tropical Medicine and Global Health, Nagasaki University, Nagasaki, Japan; 29 Department of Epidemiology and Preventive Medicine, School of Public Health and Preventive Medicine, Monash University, Melbourne, Australia; 30 Climate, Air Quality Research Unit, School of Public Health and Preventive Medicine, Monash University, Melbourne, Australia; 31 Centre for Statistical Methodology, London School of Hygiene & Tropical Medicine, London, UK; 32 Department of Environmental Health, T.H. Chan School of Public Health, Harvard University, Boston, USA; 33 Facultad de Ciencias Sociales, Instituto de Investigaciones Gino Germani, Universidad de Buenos Aires, Buenos Aires, Argentina; 34 Department of Public Health, Universidad de los Andes, Santiago, Chile; 35 Department of Environmental Health, School of Public Health, Fudan University, Shanghai, China; 36 Department of Environmental Health, University of São Paulo, São Paulo, Brazil; 37 Center for Environmental and Respiratory Health Research (CERH), University of Oulu, Oulu, Finland; 38 Medical Research Center Oulu (MRC Oulu), Oulu University Hospital and University of Oulu, Oulu, Finland; 39 Institute of Epidemiology, Helmholtz Zentrum München-German Research Center for Environmental Health (GmbH), Neuherberg, Germany; 40 IBE-Chair of Epidemiology, Ludwig-Maximilians-Universität (LMU), Munich, Germany; 41 Department of Physical, Chemical and Natural Systems, Universidad Pablo de Olavide, Seville, Spain; 42 Department of Hygiene, Epidemiology and Medical Statistics, National and Kapodistrian University of Athens, Athens, Greece; 43 School of Population Health and Environmental Sciences, King’s College, London, UK; 44 Faculty of Geography and Environmental Sciences, Hakim Sabzevari University, Sabzevar, Khorasan Razavi Iran; 45 Department of Epidemiology, Lazio Regional Health Service, Rome, Italy; 46 Department of Global Health Policy, Graduate School of Medicine, The University of Tokyo, Tokyo, Japan; 47 Department of Global Environmental Health, Graduate School of Medicine, University of Tokyo, Tokyo, Japan; 48 Department of Environmental Health, National Institute of Public Health, Cuernavaca, Morelos Mexico; 49 National Agency for Public Health of the Ministry of Health, Labour and Social Protection of the Republic of Moldova, Chișinău, Republic of Moldova; 50 National Institute for Public Health and the Environment (RIVM), Centre for Sustainability and Environmental Health, Bilthoven, The Netherlands; 51 Norwegian Institute of Public Health, Oslo, Norway; 52 School of Tropical Medicine and Global Health, Nagasaki University, Nagasaki, Japan; 53 Centro de Investigação em Saúde Pública, Escola Nacional de Saúde Pública, Universidade NOVA de Lisboa, Lisbon, Portugal; 54 Faculty of Geography, Babes-Bolay University, Cluj-Napoca, Romania; 55 Graduate School of Public Health, Seoul National University, Seoul, Republic of Korea; 56 School of the Environment, Yale University, New Haven, USA; 57 Department of Statistics and Computational Research, Universitat de València, València, Spain; 58 Ciberesp, Madrid, Spain; 59 Department of Public Health and Clinical Medicine, Umeå University, Umeå, Sweden; 60 Swiss Tropical and Public Health Institute, Basel, Switzerland; 61 University of Basel, Basel, Switzerland; 62 Environmental and Occupational Medicine, and Institute of Occupational Medicine and Industrial Hygiene, National Taiwan University (NTU) and NTU Hospital, Taipei, Taiwan, ROC; 63 National Institute of Environmental Health Science, National Health Research Institutes, Zhunan, Taiwan, ROC; 64 NTU Hospital, Taipei, Taiwan, ROC; 65 Department of Quantitative Methods, School of Medicine, University of the Republic, Montevideo, Uruguay; 66 Institute of Research and Development, Duy Tan University, Da Nang, Vietnam; 67 Department of Environmental Health, Faculty of Public Health, University of Medicine and Pharmacy, Ho Chi Minh City, Vietnam

**Keywords:** Environmental health, Epidemiology

## Abstract

Epidemiological analyses of health risks associated with non-optimal temperature are traditionally based on ground observations from weather stations that offer limited spatial and temporal coverage. Climate reanalysis represents an alternative option that provide complete spatio-temporal exposure coverage, and yet are to be systematically explored for their suitability in assessing temperature-related health risks at a global scale. Here we provide the first comprehensive analysis over multiple regions to assess the suitability of the most recent generation of reanalysis datasets for health impact assessments and evaluate their comparative performance against traditional station-based data. Our findings show that reanalysis temperature from the last ERA5 products generally compare well to station observations, with similar non-optimal temperature-related risk estimates. However, the analysis offers some indication of lower performance in tropical regions, with a likely underestimation of heat-related excess mortality. Reanalysis data represent a valid alternative source of exposure variables in epidemiological analyses of temperature-related risk.

## Introduction

In situ measurements from weather stations are often regarded as the gold-standard in epidemiological studies^1^. Though generally considered representative of the actual ambient conditions and individual’s exposure, their broader application in environmental epidemiology is often constrained by inhomogeneous records and the sparse density of meteorological stations. In addition, the geographic proximity of the measurements to the population under study is not always guaranteed. These limitations are generally more evident in low- and middle-income countries where the network of ground stations is often sparse or non-existent, or the quality control protocols are usually not standardized^2^. Moreover, even in high-income regions where historical observations are better maintained, the stations are often located outside populated areas, e.g., at nearby airports or remote weather observatories, and thus not truly representative of the exposure of populations living in large urban centres^3,4^. This further limits the potential usage of single- or multiple-monitoring stations in capturing local phenomena, such as urban heat island (UHI) effects that can enhance heat stress especially during heat waves^5^.

In recent years, data from climate reanalysis are routinely being applied as pseudo-observations in sectoral impacts assessments^6,7^. Reanalysis data products are obtained by runs of global or regional weather forecasting models under observationally constrained scenarios via data assimilation^8,9^. These products offer an immediate advantage over in situ measurements by providing consistent historical records of numerous meteorological variables, spanning the whole globe at various spatial and temporal resolutions. A number of global forecasting and research centres make their quality-controlled reanalysis data products freely available. However, their usage in health impact assessment has been limited and often been restricted to regional scale studies^10,11^.

Only a few studies to date have compared the use of climate reanalysis data by comparing estimates of epidemiological associations versus station-based observations, in particular for quantifying mortality risks associated with non-optimal temperatures^10–13^. These studies have generally found a good correlation between the two sources and similar estimates of health impact. However, these assessments were limited to single countries^10,12^ or regions^11,13^ with high-quality stations measurements, thus limiting the generalisability of the findings. More importantly, the evaluation has remained largely restricted to the inspection of temperature distributions and estimated exposure–response curves, without a comparative analysis of performance that can identify the preferable option and quantify potential biases.

**Figure 1:**
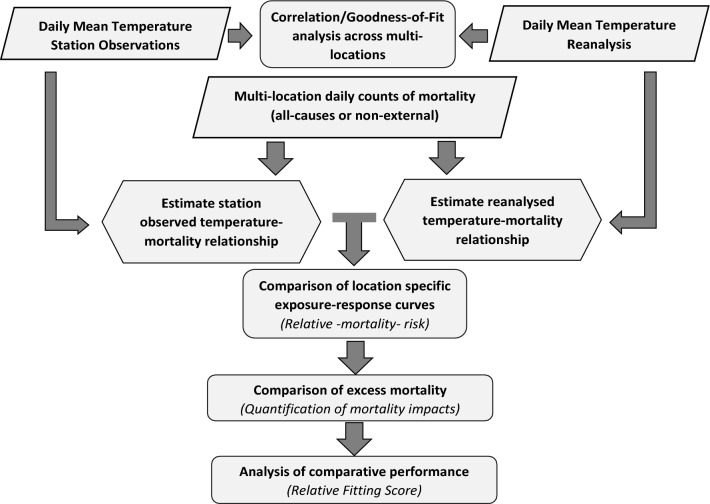
A schematic outline of the comparative analysis framework used in this study.

Here, we perform a comprehensive assessment of temperature-related mortality risks using ground weather stations observations and state-of-the-art reanalysis data across 612 cities within 39 countries over the period 1985–2019. The analysis plan is illustrated in Fig. 1. Briefly, we first systematically compared the correlation between daily temperature series derived from the two sources, then we evaluated differences in estimated exposure–response functions of temperature-mortality relationships, and finally we compared their performance using fit statistics. Our underlying objectives are to determine to what extent climate reanalysis data can be directly used in health impact analyses, and to compare their performance with ground station records in a wider multi-location multi-country setting.

### Comparison of reanalysis and station-based daily temperature time-series

We gathered daily mean temperature (°C) from station records and all-mortality series for all or non-external causes for the 612 cities from the Multi-County Multi-City (MCC) Collaborative Research Network (https://mccstudy.lshtm.ac.uk) (see “Methods”), which offers the largest epidemiological database on temperature-health associations. Daily temperature series were derived by single weather stations or averaged across multiple stations at each location. Further details of the MCC database, including the data sources and summary statistics are provided in the Supplementary Information (SI) Tables S1 and S2. These data were matched with daily mean temperature series assembled using the hourly fields of ERA5-Land climate reanalysis^14^, the most recent iteration of global reanalysis products from the European Centre for Medium Range Weather Forecasts (ECMWF) Copernicus programme that provides land-surface data at ~ 9 km resolution (see “Methods”). As sensitivity checks, we use ERA5 available at ~ 31 km resolution^9^ as a secondary dataset, whose results we report in the SI.

**Figure 2:**
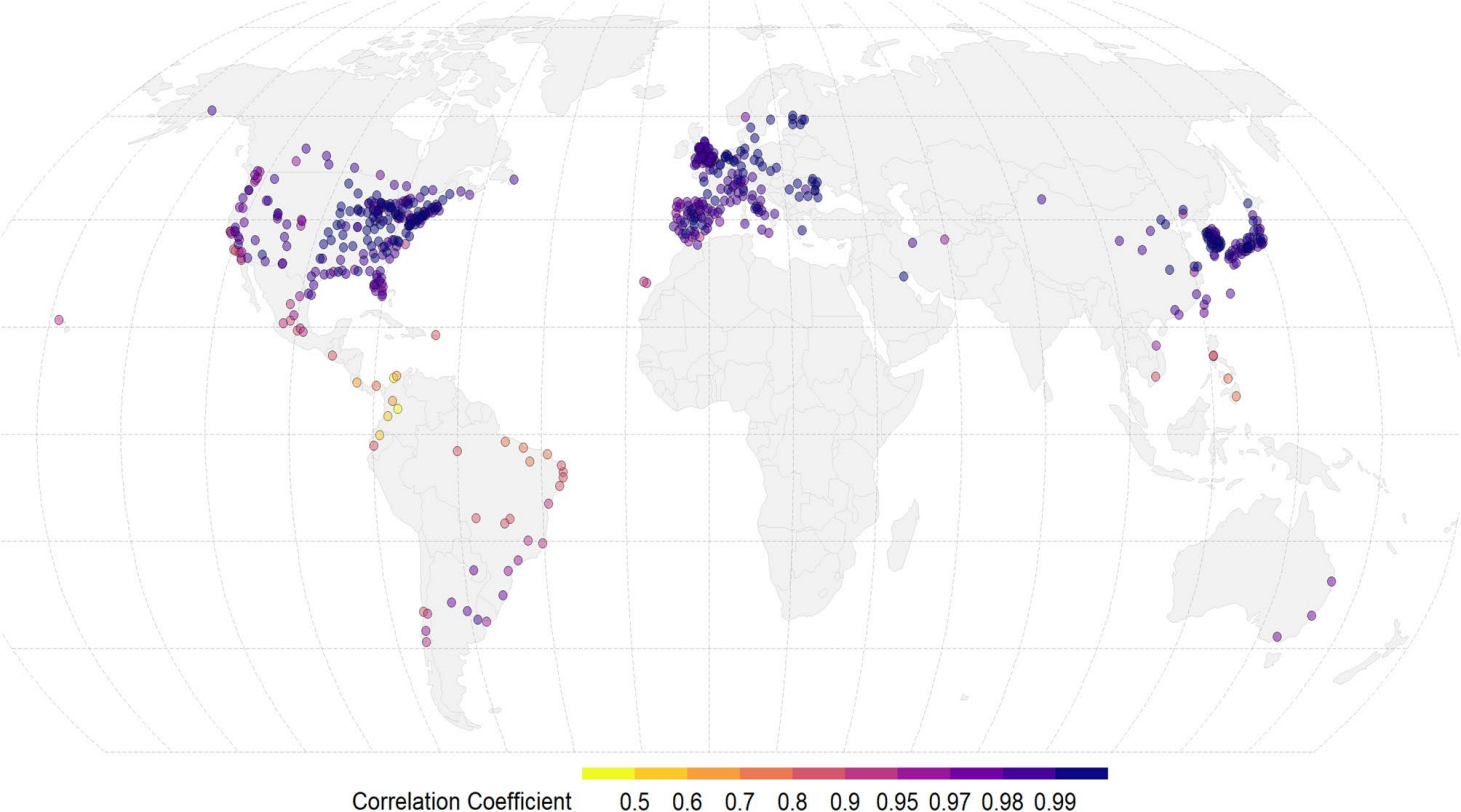
Correlation between MCC weather station and ERA5-Land daily mean temperature (°C) across the 612 locations used in the study.

We first examined the correlation between the ERA5-Land and weather stations’ temperature at each location in our study. Figure 2 shows a high correlation in most regions across the world, with a median of Pearson *r* of 0.987, and 94% of cities with a score higher than 0.9. However, while the correlation is very high in high-income regions, it drops noticeably in few locations, especially in tropical and sub-tropical countries, such as the Philippines, Central America (Costa Rica, Panama), Ecuador, Colombia, and parts of northern Brazil (see Fig. S2 in SI for the country-specific distribution of city-specific correlations).

The geographical pattern of high-correlation between the station-based and climate reanalysis temperatures is not surprising. First, the station networks and record upkeeping are better maintained in high-income regions, with more reliable weather observations^2^. Second, ground station measurements are an important component ingested in reanalysis datasets^15,16^, and are generally less reliable in areas with a sparse and lower-quality monitoring network, such as in low- and middle-income countries, or remote locations^17^. Additional goodness of fit metrics comparing ERA5-Land temperature to station observations are presented in the SI (Fig. S3, Fig. S4, Table S2).

### Comparison of estimated location-specific exposure–response associations

Using weather station and reanalysis temperature series, we next compared the exposure–response relationship that expresses the mortality risk in each of the 612 cities. We estimated the association between temperature and mortality using observed and reanalysis data through a two-stage approach widely employed in multi-location studies in environmental epidemiology^11,18–22^ (see “Methods”). In brief, the framework incorporates first-stage time-series regressions and second-stage multivariate multilevel meta-analysis to estimate the location-specific exposure–response curves reporting the relative risk (RR) at each temperature compared to a minimum mortality value (MMT).

**Figure 3:**
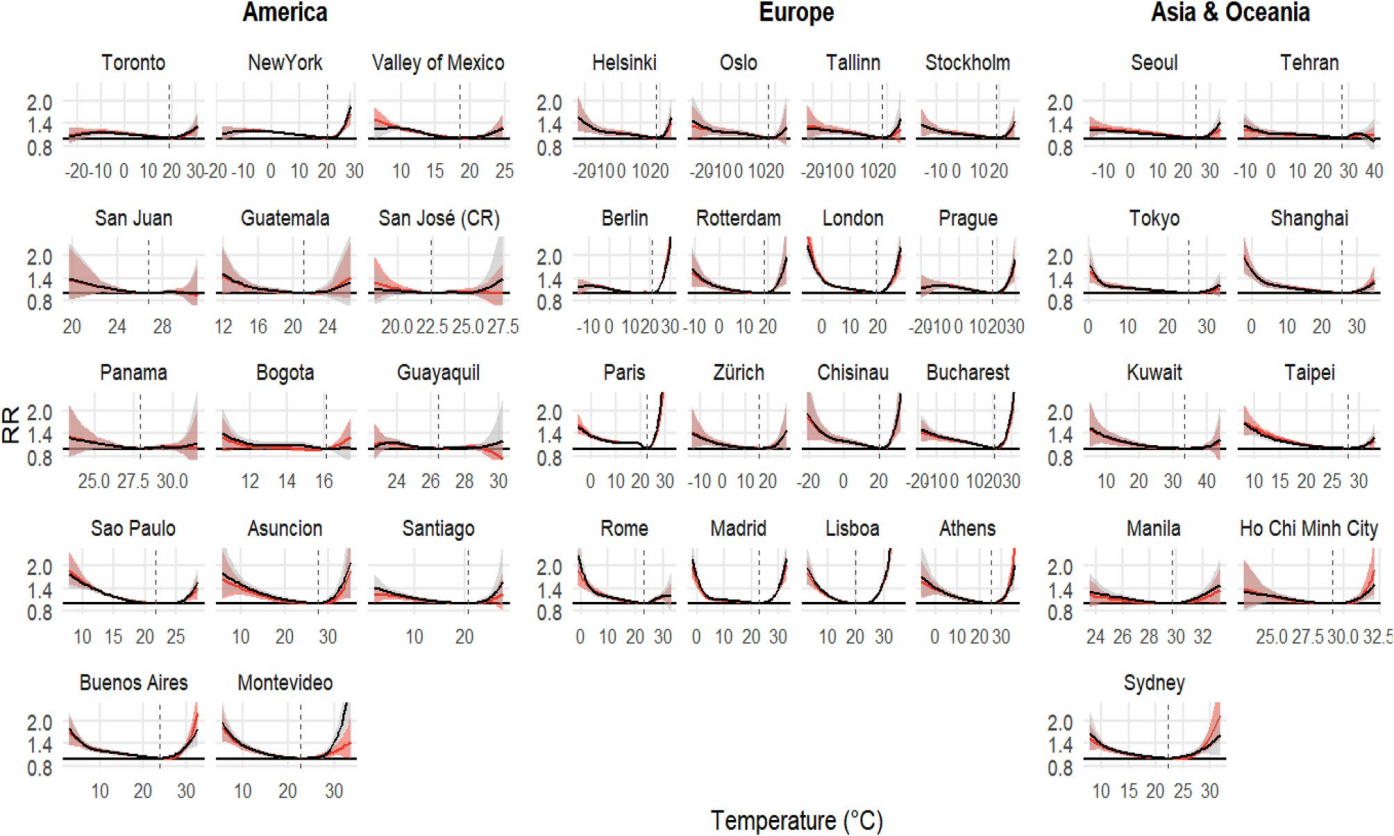
Overall cumulative exposure–response associations in selective cities representative of the 39 countries (station observations—black and ERA5-Land—red, with 95% confidence intervals (CI)—shaded, see “Methods”). Exposure–response associations as best linear unbiased prediction (BLUP, see “Methods”) using the distribution drawn from station temperature. Dashed vertical grey lines are the minimum mortality temperatures (MMTs). *RR* relative risk.

Figure 3 shows city-specific estimates of the overall cumulative exposure–response curves for a selection of cities in the 39 countries, using station observations and ERA5-Land series. An inspection of the exposure–response curves indicates overall a strong agreement between heat- and cold-related risks estimated using the two temperature series across several regions of the world. However, there are some notable exceptions. Cities in South-American countries (Argentina, Chile, Colombia, Costa Rica, Ecuador, Paraguay, and Uruguay) and South-East Asia (Philippines and Vietnam) show a noticeable divergence in heat-related RR from station observations and ERA5-Land, especially for very high temperatures. In high-income countries, the estimated RR for high temperatures shows divergence only for Sydney (Australia), and to a lesser extent in Seoul (South Korea), Tallinn (Estonia) and Athens (Greece).

**Figure 4:**
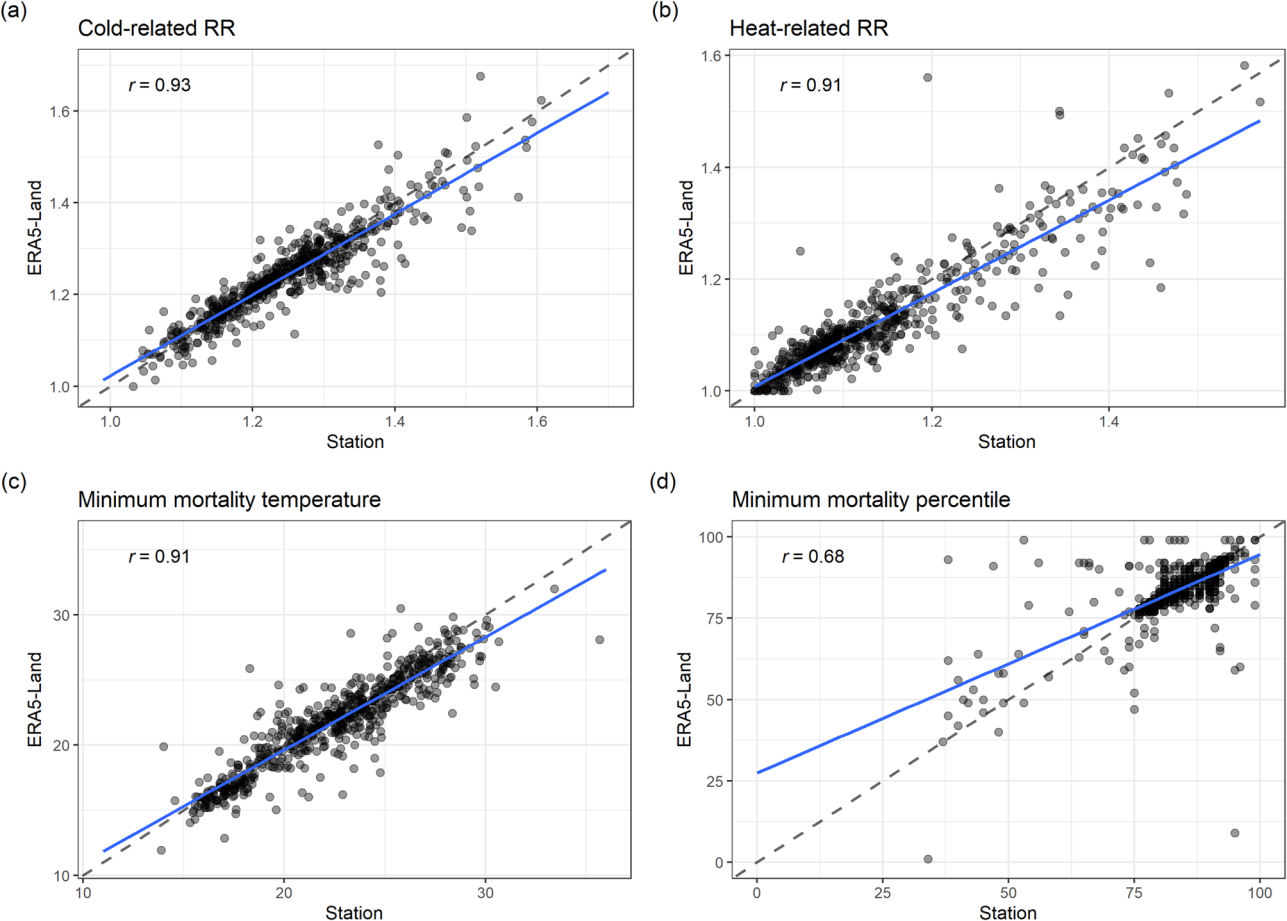
Scatterplots of: (**a**) and (**b**) cumulative relative risks (RRs) at the 1st and the 99th percentile respectively; (**c**) Minimum mortality temperature (MMT) and (**d**) Minimum mortality percentile (MMP). The RRs, MMP and MMT are based on the respective station and ERA5-Land temperatures of the best linear unbiased predictions (BLUPs) for individual cities. Blue lines and the r values represent the linear regression trend and the Pearson correlation coefficient of compared variables, respectively. The dashed black line represents the 1:1 line.

Focusing on the lower and upper bounds of the temperature distribution, the general pattern in Fig. 3 suggests that in contrast to the heat-related risks, the two sets of exposure–response curves in the 39 selected large cities agree better for cold-related risks. The pattern is similar when the exposure risks for the 1st and 99th percentile temperatures across all locations are investigated using the two temperature sources (Fig. 4, top panel), with 325 (~ 54%) locations and 395 (~ 65%) locations showing higher cold- and heat-related RRs, respectively, when using ground station data, though with an overall high correlation (r > 0.9). Additionally, across majority of the locations, the MMT and to a lesser extent, the minimum mortality percentile (MMP) from both station observed and ERA5-Land derived estimates agree well (Fig. 4 bottom panel and Table S2), with the MMT estimated using station data marginally different from the corresponding estimates derived using ERA5-Land.

### Excess mortality due to heat and cold

**Figure 5:**
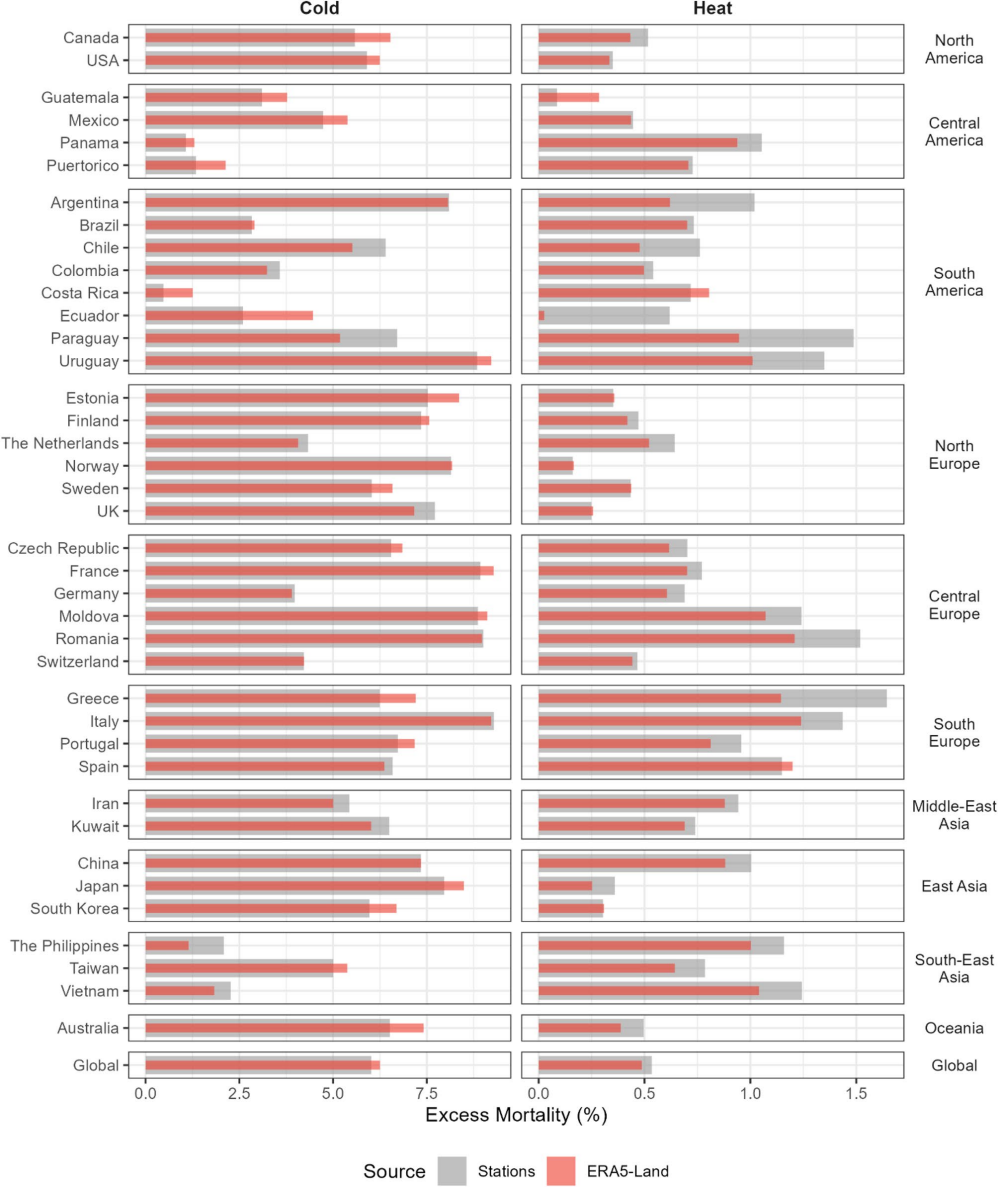
Fraction of all-cause excess mortality (%) due to cold and heat by countries and all 612 locations (Global) estimated using station observations (gray) and ERA5-Land (red). The bar plots represent the excess deaths. The 95% empirical confidence intervals (eCI) computed using Monte Carlo simulations (see “Methods”) are reported in Table S3 in the “Supplementary material”. The range of x-axes are different in the two panels.

Utilising the location-specific exposure–response relationships, we next computed the heat- and cold-related excess mortality using the station observation and reanalysis temperature. Excess mortality here is defined as the additional number of deaths and the related fraction due to heat and cold^23,24^ (see “Methods”). In brief, the excess deaths are computed for each day of the series depending on the RR associated to the daily temperature and the observed mortality, and the total is given by the sum of the daily contributions separately for the cold and hot range (below and above the MMT, respectively). The fraction is computed using the total number of deaths across the series. Figure 5 shows the estimated excess mortality fraction by country as separated components due to cold and heat derived using station observed and ERA5-Land temperatures, for each country grouped by regions (see Tables S2 and S3 in the SI for further details at the city, country, regional, climate zone and global levels).

In general, across most countries, the estimates of the excess mortality are very similar, with a global-level excess of 0.53% (95% eCI 0.50–0.56) versus 0.49% (0.43–0.53) for heat, and 6.02% (5.80–6.18) versus 6.25% (6.05–6.41) for cold, from ground stations and ERA5-Land data, respectively (‘Global’ in Fig. 5 and Table S3). These percentages correspond to 357,729 (95% eCI 335,138–376,498) versus 326,032 (288,069–357,247) for heat, and 4,030,793 (3,880,068–4,137,579) versus 4,186,014 (4,051,321–4,293,311) for cold. However, similar to the analysis of the RR, estimates of heat-related excess mortality were marginally higher for ground stations in a number of countries (Fig. 5, right panel). Specifically, the differences in heat-related excess mortality are consistent with the pattern identified in the analysis of correlation and RRs, with generally lower estimates from ERA5-Land data in regions with lower correlation with ground stations, for instance countries in South America and South-East Asia. Additionally, the same pattern is also observed in some other countries where higher correlations were noted in Fig. 2, such as Paraguay, Uruguay, Greece, and Romania, though the differences in the heat-related excess mortality here could generally be influenced by the limited sample size in our study (1–4 cities, Table S1). Focusing on the cold-related excess mortality (Fig. 5 and Table S3), the estimates from the two data sources compare remarkably better, with the ERA5-Land in some of the above countries instead showing marginally higher excess mortality than the weather station (e.g., Panama, Uruguay), and some other high-income countries such as Australia, Canada and Japan revealing a similar pattern.

### Analysis of comparative performance

**Figure 6:**
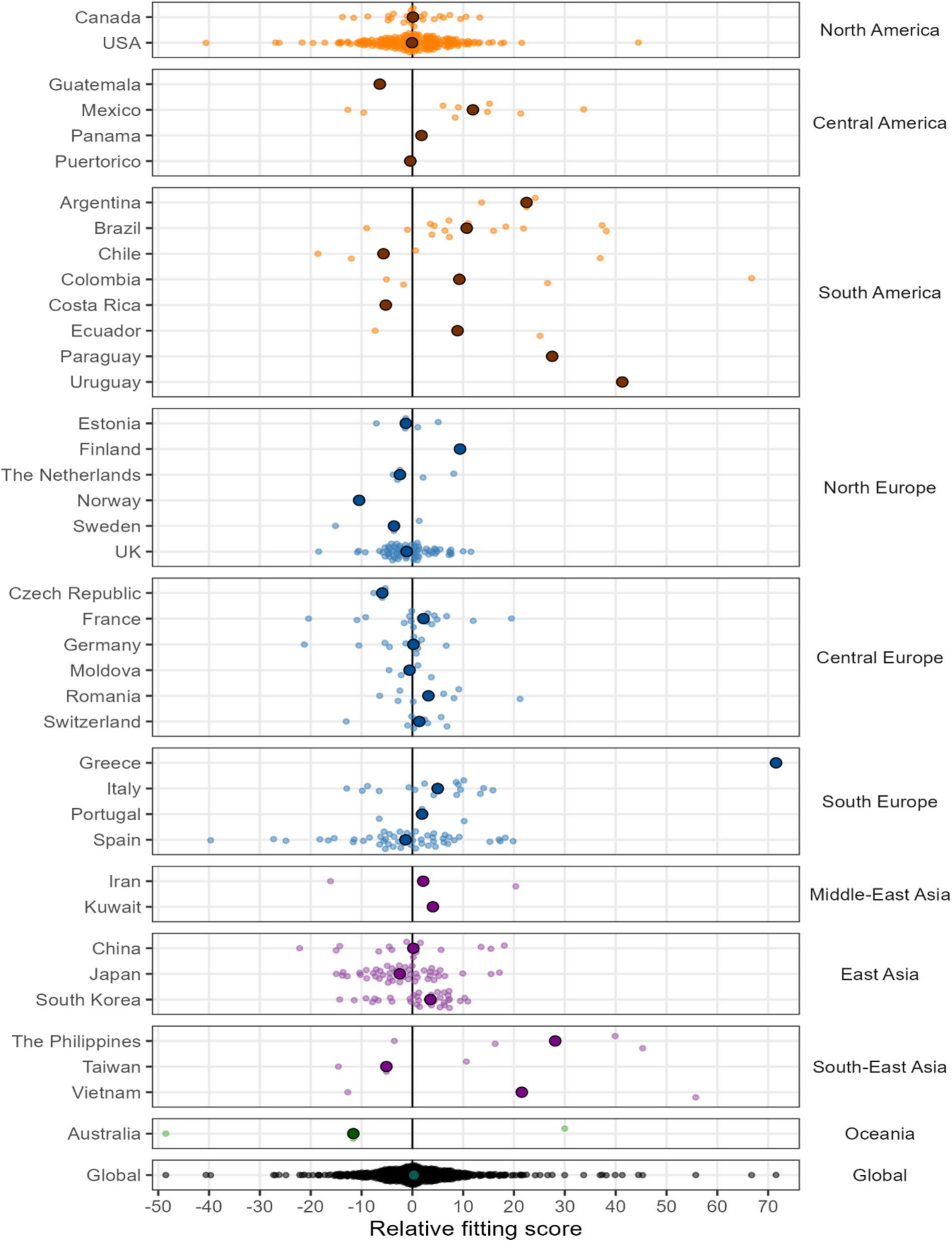
Relative fitting score (RFS) for station observations and ERA5-Land by country. A negative score indicates a better performance of the model based on ERA5-Land temperature relative to the model fitted using station temperature at a location. The shaded circle in each country panel depicts the median RFS. ‘Global’ implies all 612 locations used in the study.

To facilitate interpretation on the assessment of the two temperature data sources across geographic regions, we developed a metric to measure their comparative performance. Specifically, we defined the relative fitting score (RFS) as the difference in the quasi version of the Akaike information criterion (qAIC), which in turn is a statistic related to the goodness-of-fit of the data (see “Methods”). A negative RFS would indicate better predictive skills (i.e., lower qAIC) for the model fitted using ERA5-Land temperature, and conversely a positive RFS indicates that the use of weather station temperature improved the model’s performance (Methods). The results of RFS plotted in Fig. 6 as distribution of values by countries and globally facilitates visualization across locations/regions where each temperature data source is more predictive of mortality risks (see Fig. S5 for the map of RFS, and Table S4 for a summary of the RFS by countries).

Focusing on Fig. 6 and Fig. S5, both ERA5-Land and station observations perform likewise in majority of the locations in Europe, where actually some countries show a marginally better predictive skill for ERA5-Land. For the rest of the world, a similar pattern of neutrality in the predictive skill can be noted for the high- and upper-middle income regions (e.g., Canada, USA, Japan, China and Taiwan), with the number of locations near-equally distributed on either side of the vertical line (see Table S4 for a summary). For the remaining regions that are broadly low- and middle-income countries, the pattern suggests a better predictive skill for the station observations in South America and South-East Asia. Summarising across all locations (‘Global’ in Fig. 6 and Table S4), the predictive skill is almost balanced between the two temperature data sources, with the RFS higher for ERA5-Land in 292 cities and for weather stations in 320 cities, respectively.

### Limitations of the study and scope for further research

Our study uses ERA5-Land, a global atmospheric product that represents the last iteration of ECMWF family of reanalysis. While ERA5-Land resolves meteorological fields at the highest spatial resolution (~ 9 km) among other global reanalysis products, expanding our comparative analyses to a broader suite of datasets could enable a further systematic evaluation of the relative strengths and weaknesses of using reanalysis datasets in environmental epidemiology, an objective left for future studies. In addition, while the data gathered by the MCC network used in our study includes various types of geographical areas, we restricted our analysis to cities, as associating gridded data even at finer resolution risks of introducing aggregation bias^2^. A similar comparative analysis by estimating the exposure–response function at wider spatial scales (e.g., counties, districts, federal units) is left for future research. In spite of the large number of locations used in our study, our results for a few countries where the mortality data are restricted to limited locations (Table S1) could remain sensitive to interpretation. Efforts are ongoing with the MCC network to ingest both weather station and mortality data for a wider network of locations in such countries, as well as in countries where till date the data have remained inaccesibile (e.g., in Africa). Such efforts are expected to fill further gaps in research by facilitating similar assessments in health impact.

The primary motivation of our study was to examine the suitability of climate reanalysis for assessing temperature-related mortality, in a manner that would facilitate rapid application of gridded reanalysis products with location-specific counts of mortality in environmental epidemiology. We therefore followed the standard practice of utilising the location’s center point to extract the gridded ERA5-Land data^10,11^ (“Methods”). Noting other approaches such as the area- or the population-weighting of meteorological fields^25^ that are more commonly applied when air pollutants are the exposure variables^13^, our approach is unlikely to produce substantial variations in the temperature-mortality associations when using the high-resolution data from the recent iteration of ERA5 products, a finding highlighted in a recent study^13^.

Moreover, following literature^20,24,26–29^, our study focuses on temperature as the environmental exposure variable of interest. An added advantage of reanalysis data over ground station records is the wider availability of essential climate variables (ECV), such as wind, solar radiation, and relative humidity. ECV are commonly used to assemble thermal discomfort indices also referred to as measures of health stress, such as the apparent temperature (AT)^30,31^, the wet-bulb (WBT)^32^ and wet-bulb globe (WBGT)^33^ temperature, and the universal thermal comfort index (UTCI)^34^. However, these indices are less frequently employed to examine their relationship with mortality (e.g., ERA5-based UTCI)^11^, and previous epidemiological analyses showed little evidence of a better predictive performance of such composite indices in predicting mortality^35,36^. Nonetheless, similar comparative analyses between reanalysis and station recorded ECV (where available) are also recommended.

Finally, several location-specific factors could explain the divergence in the reanalysis and station observations derived temperature-mortality relationship, especially in the low/high temperature extremes, across the 612 locations in our study. For instance, geographical and economic factors, such as proximity of the location to a coast or hilly terrains, investments in the upkeep and maintenance of the meteorological infrastructures; and other social demographics (e.g., varying urban population density across the cities examined in our study), could likely be some of the important determinants shaping the differences in exposure–response relationships between the two temperature sources. A detailed investigation into these falls outside the scope of the present study and is left as a topic for future research.

## Discussion

Existing studies assessing the temperature-mortality associations in a large multi-location setting often employ in situ measurements^18,19,27^, or their statistically interpolated gridded counterparts^13,37^. The aim of our study was to demonstrate that climate reanalysis can potentially provide reliable surrogates of temperature for global scale epidemiological studies, thereby offering an alternative source of exposure variables. Here we evaluated the performance of temperature gathered from the ERA5-Land atmospheric reanalysis dataset, across multi-locations stratified by geographical regions, accounting for low-, middle- and high-income countries, with varying density of station records, thus providing a comprehensive and globally representative picture. To our knowledge, our study provides the first valuable insights on the suitability of current generation of quality-controlled global reanalysis datasets in a multi-location, multi-country framework, spanning five inhabited continents. By quantifying the mortality impacts from weather station and reanalysis data and performing a systematic analysis of their comparative performance, our study goes beyond published studies that limited their analyses to the exposure–response curves^10,11^, and for specific regions (Spain^10^, Europe^11^ and United States^12^). Moreover, our study is the first to examine ERA5-Land for temperature-mortality associations at a global scale, the high spatial resolution of its gridded meteorological fields (~ 9 km) documented to represent land surface variables better^14^.

Our analyses reveal that ambient temperature drawn from ERA5-Land is generally suitable to elucidate the effects of thermal stress on mortality. Importantly, in line with the previous two regional scale studies (Spain^10^ and Europe^11^) that found largely comparable exposure–response curves from weather station and ERA5 data, our analyses using ERA5-Land revealed a similar pattern across 612 locations in 39 countries. Our results are also comparable to the earlier well-established global multi-location studies based on station records^19,24,26,27^ that document similar magnitude of excess mortality from heat and cold exposures across the full range of temperatures. However, it must be pointed out that while largely in agreement, the heat-related excess mortality estimated using ERA5-Land is marginally lower compared to those estimated using station observations. The magnitude of such differences is amplified in low- and middle-income countries generally in the tropics. Such differences, even in locations with relatively high correlations between the two temperature data sources, seems to suggest a sub-optimal performance of reanalysis data in identifying extremely high temperatures in specific regions, that in turn can lead to an underestimation of the health impacts of heat. While not investigated here and left as scope for further research, recent studies^14,38^ have documented improved ERA5-Land derived daily maximum temperatures compared to earlier reanalysis products, though marginal errors relative to the corresponding station observed daily maximum temperatures remain, which could explain the under-estimated excess mortality related to heat in our findings.

In summary, our study provides the first comprehensive comparison between reanalysis and station-based data for modelling temperature-mortality associations at a global scale. Our statistical assessments have a potential to inform the wider research community about the relative performance of meteorological variables and indices derived from reanalysis data in wider epidemiological analyses. It must be emphasized that the objective of our study is not to advocate the replacement of station observed data by reanalysis datasets for global-scale environmental epidemiological analyses. Systematic errors are known to exist in reanalysis products and ERA5-Land is no exception to these^11^. Yet, the advantages of homogenized, freely accessible, and frequently updated data covering all regions at high spatio-temporal resolutions, have a potential to make reanalysis products suitable for use as environmental exposure variables, especially in regions such as large swaths of Africa, where observations from sparse ground station network can present significant limitations. The consistent spatio-temporal coverage also makes reanalysis data attractive for quantifying population attributable fractions, an indicator important for health planners and policy makers. Wherever possible though, we recommend using both station observed and reanalysis data for a better quantification of uncertainty in results emanating from the source of input meteorological variables.

## Methods

All analyses in this study were done with R software (version 4.1.0)^39^ using packages dlnm^40^ and mixmeta^41^, applied and discussed in earlier studies^24,41–43^. All graphics in the study including in the supplementary material (except Fig. 1 which was created in Microsoft Word) have been generated using R package ggplot2 (ver 3.3.5)^44^. The replication code for all analyses performed in this study is available upon request from the corresponding authors. In addition, a number of reproducible examples are included on the personal website of the senior author (http://www.ag-myresearch.com/r-code.html). The mortality series used in the study consist of data aggregated over large geographical areas, all ages, and all or non-external causes. The data was originally provided by statistical authorities in each country from separate data requests and were part of administrative databases including completely anonymised information for which so the informed consent is not required.

### Mortality and weather station data

Daily counts of mortality and station observed mean temperature for 612 sites across 39 countries (Table S1) are drawn from the Multi-Country Multi-City (MCC) Collaborative Research Network (http://mccstudy.lshtm.ac.uk). MCC is a result of an international partnership between research teams producing epidemiological evidence on the association between weather and health across the globe. The MCC Network has been instrumental in developing state-of-the-art methods in environmental epidemiology, as well as in assembling the largest database on weather and health. The data, used in several earlier studies^20,27–29,45^, facilitates continent-wide analysis of environmental stressors and mortality. The data used in the present study consists of location-specific counts of daily mortality from all causes or non-external causes only (International Classification of Diseases, ICD-9: 0–799; ICD-10: A00–R99) obtained from local authorities within each country or region, and the daily mean temperature (°C) gathered from the local weather stations (Table S1). For our study, the analysis included 68,357,187 deaths across all 612 locations from 39 countries in overlapping periods between 1985 and 2019 (Table S2). Though the MCC network also includes mortality and weather station data for wider regions (e.g., provinces), assigning gridded reanalysis fields to the geographical boundaries of varying shapes and sizes would require area weighting and aggregation of the meteorological variable. To minimize a potential aggregation bias emanating from such spatial interpolation and averaging^2^, we omitted data from 141 such wider regions spanning four countries, and instead chose to restrict our analysis to the 612 urban areas. It is also worth emphasizing that the observed daily mean temperature used as the exposure index is drawn from city-specific central monitoring stations gathered by the MCC network in each country. Our study therefore largely benefits from this detailed spatially explicit weather exposure which becomes difficult to achieve when using publicly available global daily station data, such as the Global Historical Climate Network-Daily (GHCN-D)^46^ and the UK Met Office Hadley Centre Integrated Surface Database (HadISD)^47,48^ that often lack consistent spatio-temporal coverage to construct location-specific time series.

Since the MCC data are available for different time intervals across countries, our multi-location daily time series span different time periods between 1985 and 2019, with the shortest being 4 years (2013–2016) for Panama, and the longest being 34 years (1985–2018) for Norway and Portugal. Further details on the individual location-specific sources of mortality and station data included in the MCC network are documented in Refs.^19,24^.

### Global climate reanalysis datasets

We utilised hourly 2-m air temperature from ERA5-Land^14,49^ made available by the European Centre for Medium-Range Weather Forecasts (ECMWF) through the Copernicus Climate Change Service (C3S) Climate Data Store (https://climate.copernicus.eu/climate-reanalysis)^50^. ERA5-Land resolves meteorological fields at 0.09° (~ 9 km) gridded resolution, the finest spatial resolution across all global reanalysis data products that are currently available. As a secondary reanalysis data source, we utilised the hourly 2-m air temperature from ERA5^51,52^ that is available at 0.25° (~ 31 km) gridded resolution (results are presented in the SI).

ERA5-Land is the most recent atmospheric reanalysis from the ECMWF family of reanalysis datasets^14,51^ covering the land surface of the entire globe. It ingests more data sources along with the latest version of the Integrated Forecasting System, incorporates modern parameterizations techniques, and is till date the most advanced reanalysis^14,53^. It resolves many atmospheric and land-surface parameters in near real-time, thus offering a large number of meteorological parameters from 1950 to near-present day. In addition, the hourly time resolution enables an improved evolution of day-to-day weather systems^8,9^. The high spatial resolution in ERA5-Land is achieved by driving the model with statistically downscaled meteorological forcing^14,51^ and a lapse rate correction. ERA5-Land thus offers advantages with a better representation of the land surface processes compared to other current generation of reanalysis data products^14^.

We matched the reanalysis grid cell where each MCC location’s centre point was located and aggregated the hourly temperature fields to daily averages for years 1985–2019, taking care to recode the reanalysis daily values as missing where the corresponding station temperature was not available. Computation of the daily averages as 24-h average is also consistent with the daily average station recorded temperature across majority of the locations in the MCC network (Table S1). The resulting MCC-reanalysis daily time-series facilitated a detailed comparative analysis of the temperature-mortality relationship with its station observations counterpart as described in the ‘Two-stage modelling framework’ below.

### Description of the epidemiological framework

#### Two-stage modelling framework

We applied the well-established two-stage modelling framework^11,18–20,42,43^ to model the station observed and reanalysis temperature-mortality associations, across the 612 locations covering a wide range of climates and including low- and middle-income countries. Location specific temperature-mortality associations were estimated through time-series analyses with quasi-Poisson regression, with distributed lag non-linear models and multivariate meta-regression, using R packages dlnm^40^ and mixmeta^41^. Multivariate meta-analysis represents a useful analytical tool for pooling complex associations through a two-stage procedure^22^. The flexible modelling framework allows for non-linear/lagged responses, separation of effects due to cold/heat and moderate/extreme temperature, and heterogeneity of estimates at various geographical levels.^21,24,41^.

##### First stage

To estimate location-specific temperature-mortality associations, we performed separate time series analyses with generalized linear models using observed- and reanalysis-temperature and mortality data over the entire year in each location. We applied a quasi-Poisson regression in which a quasi-likelihood was used to scale the standard deviation of the coefficients proportionally to the observed overdispersion. We modelled using distributed-lag non-linear models (DLNMs), a class of models that can describe the complex non-linear and lagged dependencies typically found in temperature-mortality studies^54^. DLNMs account for delayed effects of time-varying exposures and quantify overall effects over a predefined lag period. Following the DLNM methodology, we modelled the bidimensional exposure-lag-response association through the combination of two functions defined within a cross-basis term. Specifically, we selected a natural cubic spline function with three internal knots at the 10th, 75th and 90th percentile of the location-specific temperature distribution to model the exposure–response curve, and a natural cubic spline function with three internal knots at equally spaced values in the log scale over 21 days of lag for the lag-response dimension. Seasonality and long-term trends were modelled with a natural cubic spline with 8 degrees of freedom (df) of time, and the model included indicator variables for the day of the week to account for intra-weekly variations in mortality. These choices that specify the cross-basis and model terms used to control for long-term and seasonal trends were based on related studies from the MCC Collaborative Research Network^19,20^. The resulting bidimensional set of coefficients from each location was then reduced across the lag dimension into the overall cumulative exposure–response curve representing the association between temperature and mortality summed across the 21 days of lag^21^.

##### Second stage

The location-specific set of reduced coefficients estimated in the first stage were then pooled in a multivariate multilevel meta-regression model^21,41^, with two nested levels of random effects defined a city and combinations of climate zones and country. This approach allows heterogeneous effects and provides improved estimates of temperature-mortality associations at city level, defined as best linear unbiased predictions (BLUPs). BLUPs borrow information across units within the same hierarchical level and can offer more accurate estimates, especially in locations with small daily mortality counts or short series. Put differently, BLUPs represent a trade-off between the location-specific association provided by the first-stage regression and the pooled association. This approach enables more robust estimates of risk ratios (RRs) in individual cities compared to location-specific models^23,24^. We also included, as fixed-effects meta-predictors, country-level gross domestic product, location-specific average temperature and interquartile range and indicators of climatic classification^55^.

We tested and quantified the presence of heterogeneity using multilevel extensions of the Cochran Q test and I^2^ statistic^22,56^. The city-specific associations defined by the BLUPs were used in the quantification of the cold- and heat-related mortality impacts.

### Excess mortality due to heat and cold

Next, we quantified the station observed and reanalysis temperature-related mortality in each location during the study period of 1985–2019 following a method described in previous works^23,24,42^. For each location-day combination, we computed the number of cold- and heat-related deaths on the basis of temperature series, daily baseline mortality and the estimated temperature-mortality association represented by the location-specific BLUPs^23^. Following earlier works^23,24,43^, we then estimated the total number of cold- and heat-related deaths in each location across the study period by summing the daily mortality contributions when the temperature on a specific day was lower (higher) than the location-specific reference temperature. This reference value corresponds to the minimum point of the BLUP curve and represents the optimal temperature value with the lowest mortality risk, often referred to as the minimum mortality temperature (MMT). We quantified the uncertainty of the estimates by generating 1,000 samples of the coefficients of the BLUPs (representing the association) through Monte Carlo simulations, assuming a multivariate normal distribution for the estimated spline model coefficients. We obtained empirical confidence intervals (eCI) corresponding to the 2.5th and 97.5th percentiles of the empirical distribution of the cold- and heat- related mortality impacts across coefficients. Finally, as elaborated in previous studies^13,23,24^, we computed the mortality fractions using the related total number of deaths as the denominator.

### Analysis of comparative fit performance (relative fitting score-RFS)

We computed the quasi-Akaike information criterion (qAIC) used in earlier studies^13,36^ to examine the ability of the two temperature series to predict all-cause mortality at each location. This approach provides a quantitative evaluation on the performance of each source (reanalysis temperature relative to the station observed data) in modelling excess mortality risks associated with non-optimal temperature. As noted earlier, since the first-stage models employing station observed and reanalysis temperature were both fitted using the same data sample (i.e., ensuring any missing observations in daily station records were also systematically omitted from the reanalysis time-series), the qAIC becomes comparable and can be used as a robust goodness of fit metric between the two sources. This statistic can be summed across countries or regions to facilitate comparisons at different geographical levels.

To facilitate an easier interpretation of the preferred model, we defined a new metric called relative fitting score (RFS), as a measure of relative fitting performance. The RFS is computed as the difference between the two qAICs:

$$RFS=qAIC\left(Reanalysis\right)- qAIC \left(Station\;Observed\right)$$

with a negative RFS indicating a superior predictive ability when using reanalysis temperature, and conversely a positive RFS suggesting a better performance of ground station observations.

## Supplementary Information

Supplementary Information 1.

Supplementary Information 2.

## Data Availability

Data from ERA5 and ERA5-Land are publicly available at https://cds.climate.copernicus.eu. Station-based temperature and mortality data were collected from participants in individual countries from meteorological and health statistics institutions. The data are often released under specific agreements that prevent them to be released publicly.

## Code availability

Code to perform the analysis and reproduce the figures in the paper is available upon request from the corresponding authors.
