# Author Correction: Comparison of weather station and climate reanalysis data for modelling temperature-related mortality

**DOI:** 10.1038/s41598-022-11769-6

**Published:** 2022-05-13

**Authors:** Malcolm N. Mistry, Rochelle Schneider, Pierre Masselot, Dominic Royé, Ben Armstrong, Jan Kyselý, Hans Orru, Francesco Sera, Shilu Tong, Éric Lavigne, Aleš Urban, Joana Madureira, David García-León, Dolores Ibarreta, Juan-Carlos Ciscar, Luc Feyen, Evan de Schrijver, Micheline de Sousa Zanotti Stagliorio Coelho, Mathilde Pascal, Aurelio Tobias, Multi-Country Multi-City (MCC) Collaborative Research Network, Barrak Alahmad, Rosana Abrutzky, Paulo Hilario Nascimento Saldiva, Patricia Matus Correa, Nicolás Valdés Orteg, Haidong Kan, Samuel Osorio, Ene Indermitte, Jouni J. K. Jaakkola, Niilo Ryti, Alexandra Schneider, Veronika Huber, Klea Katsouyanni, Antonis Analitis, Alireza Entezari, Fatemeh Mayvaneh, Paola Michelozzi, Francesca de’Donato, Masahiro Hashizume, Yoonhee Kim, Magali Hurtado Diaz, César De la Cruz Valencia, Ala Overcenco, Danny Houthuijs, Caroline Ameling, Shilpa Rao, Xerxes Seposo, Baltazar Nunes, Iulian-Horia Holobaca, Ho Kim, Whanhee Lee, Carmen Íñiguez, Bertil Forsberg, Christofer Åström, Martina S. Ragettli, Yue-Liang Leon Guo, Bing-Yu Chen, Valentina Colistro, Antonella Zanobetti, Joel Schwartz, Tran Ngoc Dang, Do Van Dung, Yuming Guo, Ana M. Vicedo-Cabrera, Antonio Gasparrini

**Affiliations:** 1 Department of Public Health, Environments and Society, London School of Hygiene & Tropical Medicine, London, UK; 2 Department of Economics, Ca’ Foscari University of Venice, Venice, Italy; 3 The Centre on Climate Change & Planetary Health, London School of Hygiene & Tropical Medicine, London, UK; 4 Forecast Department, European Centre for Medium-Range Weather Forecast (ECMWF), Reading, UK; 5 Ф-Lab, European Space Agency (ESA-ESRIN), Frascati, Italy; 6 Department of Geography, University of Santiago de Compostela, Santiago de Compostela, Spain; 7 CIBER de Epidemiología y Salud Pública (CIBERESP), Madrid, Spain; 8 Institute of Atmospheric Physics of the Czech Academy of Sciences, Prague, Czech Republic; 9 Faculty of Environmental Sciences, Czech University of Life Sciences, Prague, Czech Republic; 10 Department of Family Medicine and Public Health, University of Tartu, Tartu, Estonia; 11 Department of Statistics, Computer Science and Applications ‘G. Parenti’, University of Florence, Florence, Italy; 12 Shanghai Children’s Medical Center, Shanghai Jiao Tong University School of Medicine, Shanghai, China; 13 School of Public Health, Institute of Environment and Population Health, Anhui Medical University, Hefei, China; 14 School of Public Health and Social Work, Queensland University of Technology, Brisbane, QLD Australia; 15 Center for Global Health, School of Public Health, Nanjing Medical University, Nanjing, China; 16 Air Health Science Division, Health Canada, Ottawa, ON Canada; 17 School of Epidemiology and Public Health, University of Ottawa, Ottawa, ON Canada; 18 Department of Environmental Health, Instituto Nacional de Saúde Dr Ricardo Jorge, Porto, Portugal; 19 EPIUnit—Instituto de Saúde Pública, Universidade do Porto, Porto, Portugal; 20 The Joint Research Center (JRC), European Commission, Seville, Spain; 21 The Joint Research Center (JRC), European Commission, Ispra, Italy; 22 Graduate School of Health Science, University of Bern, Bern, Switzerland; 23 Institute of Social and Preventive Medicine, University of Bern, Bern, Switzerland; 24 Oeschger Center for Climate Change Research, University of Bern, Bern, Switzerland; 25 Faculty of Medicine, University of São Paulo, São Paulo, Brazil; 26 Santé Publique France, Department of Environmental and Occupational Health, French National Public Health Agency, Saint Maurice, France; 27 Institute of Environmental Assessment and Water Research (IDAEA), Spanish Council for Scientific Research (CSIC), Barcelona, Spain; 28 School of Tropical Medicine and Global Health, Nagasaki University, Nagasaki, Japan; 29 Department of Epidemiology and Preventive Medicine, School of Public Health and Preventive Medicine, Monash University, Melbourne, Australia; 30 Climate, Air Quality Research Unit, School of Public Health and Preventive Medicine, Monash University, Melbourne, Australia; 31 Centre for Statistical Methodology, London School of Hygiene & Tropical Medicine, London, UK; 32 Department of Environmental Health, T.H. Chan School of Public Health, Harvard University, Boston, USA; 33 Facultad de Ciencias Sociales, Instituto de Investigaciones Gino Germani, Universidad de Buenos Aires, Buenos Aires, Argentina; 34 Department of Public Health, Universidad de los Andes, Santiago, Chile; 35 Department of Environmental Health, School of Public Health, Fudan University, Shanghai, China; 36 Department of Environmental Health, University of São Paulo, São Paulo, Brazil; 37 Center for Environmental and Respiratory Health Research (CERH), University of Oulu, Oulu, Finland; 38 Medical Research Center Oulu (MRC Oulu), Oulu University Hospital and University of Oulu, Oulu, Finland; 39 Institute of Epidemiology, Helmholtz Zentrum München-German Research Center for Environmental Health (GmbH), Neuherberg, Germany; 40 IBE-Chair of Epidemiology, Ludwig-Maximilians-Universität (LMU), Munich, Germany; 41 Department of Physical, Chemical and Natural Systems, Universidad Pablo de Olavide, Seville, Spain; 42 Department of Hygiene, Epidemiology and Medical Statistics, National and Kapodistrian University of Athens, Athens, Greece; 43 School of Population Health and Environmental Sciences, King’s College, London, UK; 44 Faculty of Geography and Environmental Sciences, Hakim Sabzevari University, Sabzevar, Khorasan Razavi Iran; 45 Department of Epidemiology, Lazio Regional Health Service, Rome, Italy; 46 Department of Global Health Policy, Graduate School of Medicine, The University of Tokyo, Tokyo, Japan; 47 Department of Global Environmental Health, Graduate School of Medicine, University of Tokyo, Tokyo, Japan; 48 Department of Environmental Health, National Institute of Public Health, Cuernavaca, Morelos Mexico; 49 National Agency for Public Health of the Ministry of Health, Labour and Social Protection of the Republic of Moldova, Chişinău, Republic of Moldova; 50 National Institute for Public Health and the Environment (RIVM), Centre for Sustainability and Environmental Health, Bilthoven, The Netherlands; 51 Norwegian Institute of Public Health, Oslo, Norway; 52 School of Tropical Medicine and Global Health, Nagasaki University, Nagasaki, Japan; 53 Centro de Investigação em Saúde Pública, Escola Nacional de Saúde Pública, Universidade NOVA de Lisboa, Lisbon, Portugal; 54 Faculty of Geography, Babes-Bolay University, Cluj-Napoca, Romania; 55 Graduate School of Public Health, Seoul National University, Seoul, Republic of Korea; 56 School of the Environment, Yale University, New Haven, USA; 57 Department of Statistics and Computational Research, Universitat de València, València, Spain; 58 Ciberesp, Madrid, Spain; 59 Department of Public Health and Clinical Medicine, Umeå University, Umeå, Sweden; 60 Swiss Tropical and Public Health Institute, Basel, Switzerland; 61 University of Basel, Basel, Switzerland; 62 Environmental and Occupational Medicine, and Institute of Occupational Medicine and Industrial Hygiene, National Taiwan University (NTU) and NTU Hospital, Taipei, Taiwan, ROC; 63 National Institute of Environmental Health Science, National Health Research Institutes, Zhunan, Taiwan, ROC; 64 NTU Hospital, Taipei, Taiwan, ROC; 65 Department of Quantitative Methods, School of Medicine, University of the Republic, Montevideo, Uruguay; 66 Institute of Research and Development, Duy Tan University, Da Nang, Vietnam; 67 Department of Environmental Health, Faculty of Public Health, University of Medicine and Pharmacy, Ho Chi Minh City, Vietnam

Correction to: *Scientific Reports* 10.1038/s41598-022-09049-4, published online 25 March 2022

The original version of this Article contained an error in Affiliation 25, which was incorrectly given as ‘Faculty of Medicine ArqFuturo INSPER, University of São Paulo, São Paulo, Brazil’. The correct affiliation is listed below.

Faculty of Medicine, University of São Paulo, São Paulo, Brazil

The original Article has been corrected.

