# Supplementary material for: Comparison of weather station and climate reanalysis data for modelling temperature-related mortality
Source: Sci Rep. 2022 Mar 25;12:5178. doi: 10.1038/s41598-022-09049-4 (PMC8956721; doi:10.1038/s41598-022-09049-4)
Supplement: Supplementary file 1 — Supplementary Information 1. [file 41598_2022_9049_MOESM1_ESM.docx]

**Supplementary Information: Comparison of weather station and climate reanalysis data for modelling temperature-related mortality**

Malcolm N. Mistry, Rochelle Schneider, Pierre Masselot, Dominic Royé, Ben Armstrong, Jan Kyselý, Hans Orru, Francesco Sera, Shilu Tong, Éric Lavigne, Aleš Urban, Joana Madureira, David García-León, Dolores Ibarreta, Juan-Carlos-Ciscar, Luc Feyen, Evan de Schrijver, Micheline de Sousa Zanotti Stagliorio Coelho, Mathilde Pascal, Aurelio Tobias, Multi-Country Multi-City (MCC) Collaborative Research Network^†^, Yuming Guo, Ana M. Vicedo-Cabrera and Antonio Gasparrini

† A full list of the authors from the Multi-Country Multi-City (MCC) Collaborative Research Network appears at the end of the supplementary information.

**List of Figures:**

**Figure S1:** Map of a**verage daily mean temperature – MCC station observations.**

**Figure S2: Correlation plot - MCC weather station and ERA5-Land daily mean temperature.**

**Figure S3:** Map of **Root Mean Square Error (RMSE) - MCC weather station and ERA5-Land daily mean temperature.**

**Figure S4: RMSE plot - MCC weather station and ERA5-Land daily mean temperature.**

**Figure S5:** Map of Relative Fitting Score (RFS) – MCC weather station and ERA5-Land.

**Sensitivity results using ERA5**

**Figure S6:** **Correlation map - MCC weather station and ERA5 daily mean temperature.**

**Figure S7: Correlation plot - MCC weather station and ERA5 daily mean temperature.**

**Figure S8:** Map of **Root Mean Square Error (RMSE) - MCC weather station and ERA5 daily mean**

**temperature.**

**Figure S9: RMSE plot - MCC weather station and ERA5 daily mean temperature.**

**Figure S10:** Overall cumulative exposure-response associations (Relative Risk) – MCC weather station and

ERA5.

**Figure S11:** – Scatter plot – MCC weather station and ERA5 cold- and heat-related relative risk (RR),

minimum mortality temperature (MMT) and minimum mortality percentile (MMP).

**Figure S12:** Fraction of all-cause excess mortality due to cold and heat – MCC weather station and ERA5.

**Figure S13:** Map of Relative Fitting Score (RFS) – MCC weather station and ERA5.

**Figure S14:** RFS plot – MCC weather station and ERA5.

**List of Tables:**

**Table S1:** MCC weather station and mortality data by countries.

**Table S2:** Detailed summary statistics (File Supplementary_Table_S2.xlsx).

- Summary statistics of mortality, temperature, goodness of fit (correlation, RMSE) and predictive skill of model (Quasi Akaike Information Criteria -qAIC-, RFS) – city level.
- MMT and MMP – city level.
- RR for cold and heat – city level.
- Excess mortality summarised – global level.
- Fraction of excess mortality (%) – global level.
- Excess mortality summarised – city level.
- Fraction of excess mortality (%) – city level.
- Excess mortality summarised – country level.
- Fraction of excess mortality (%) – country level.
- Excess mortality summarised – regional level.
- Fraction of excess mortality (%) – regional level.
- Excess mortality summarised – climate zone.
- Fraction of excess mortality (%) – climate zone.

**Table S3:** Fraction of all-cause excess mortality due to cold and heat – MCC weather station, ERA5-Land and

ERA5 – by countries.

**Table S4:** Summary of RFS by countries.


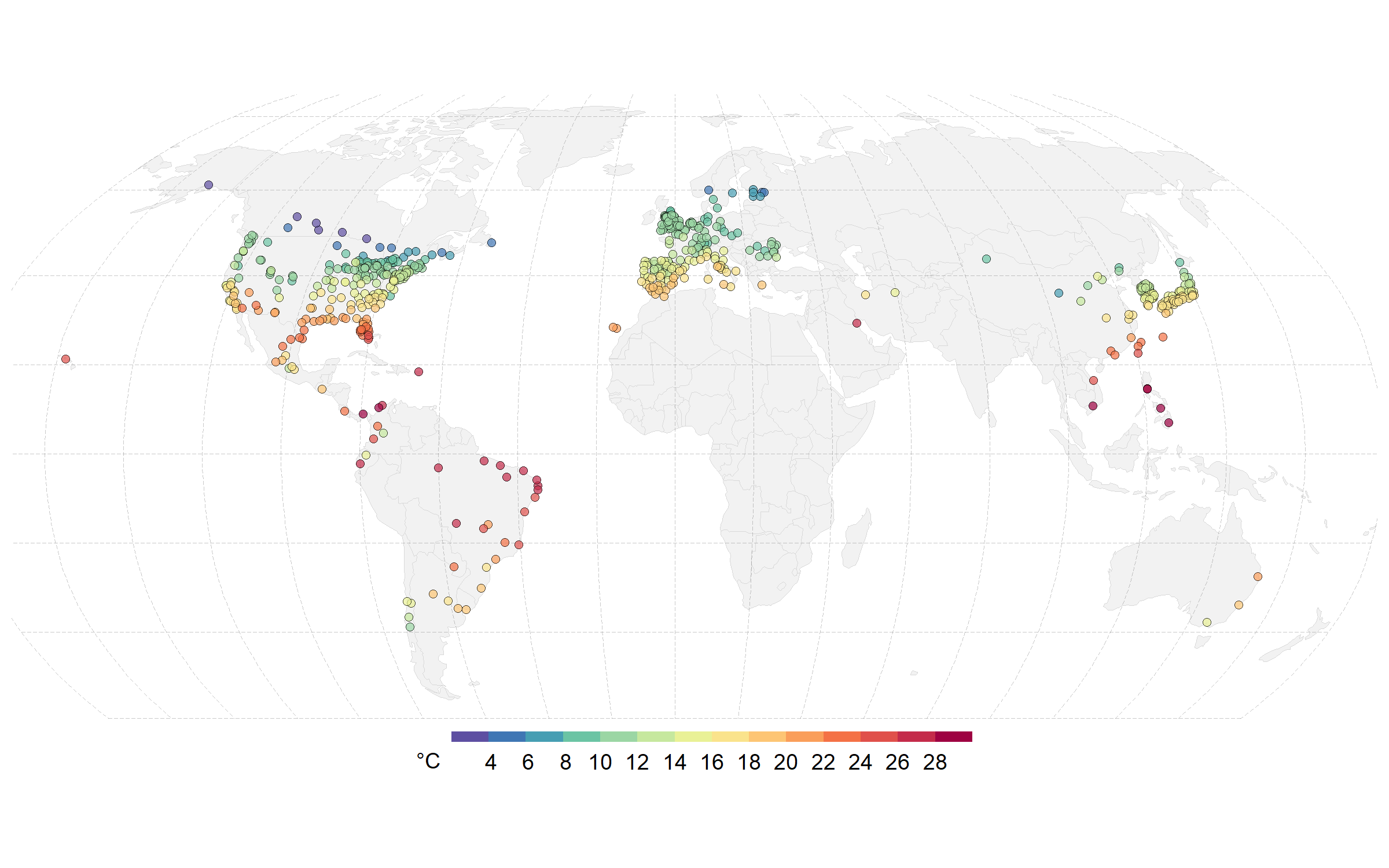


**Figure S1: Average daily mean temperature (°C) at the** 612 locations across 39 countries or territories used in this study. The average daily mean temperature is computed for the MCC data collection periods shown in Table S1.


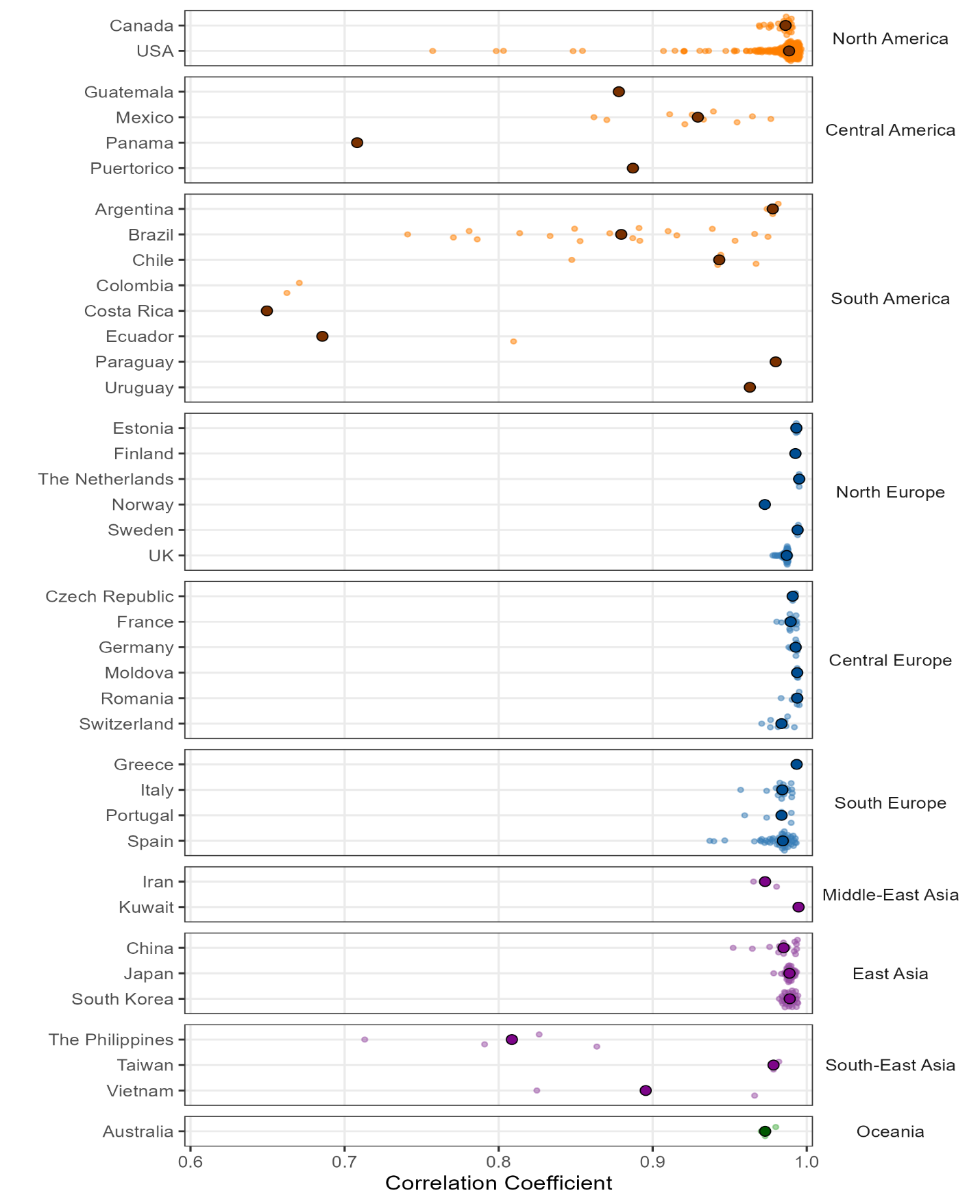


**Figure S2: Correlation between MCC weather station and ERA5-Land daily mean temperature (°C) across the 612 locations used in the study grouped by 39 countries and 10 regions. The circle in each country panel depicts the median correlation.**


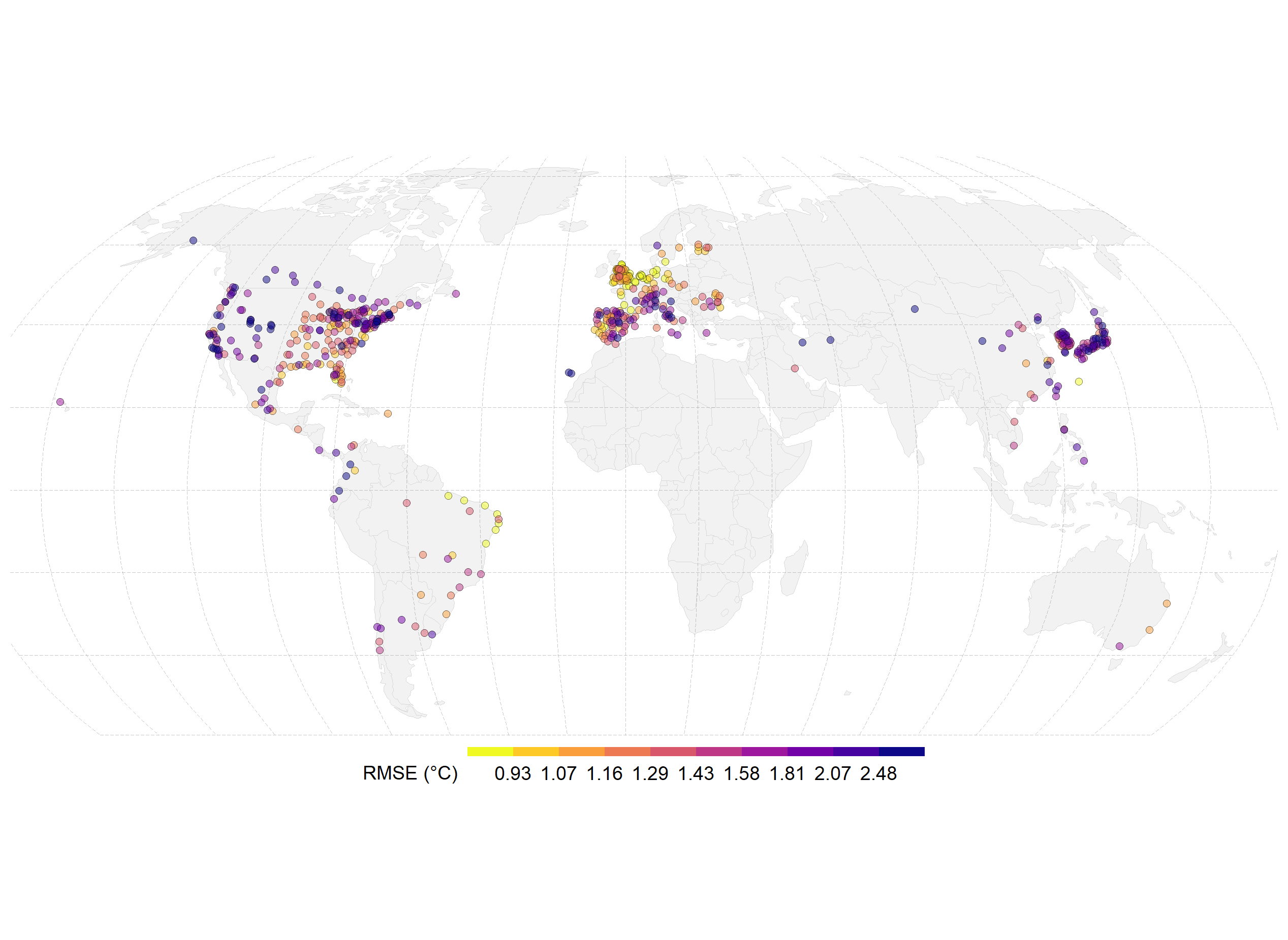


**Figure S3: Root Mean Square Error (RMSE) computed as the squared difference between MCC weather station and ERA5-Land daily mean temperature (°C) across the 612 locations used in the study.**


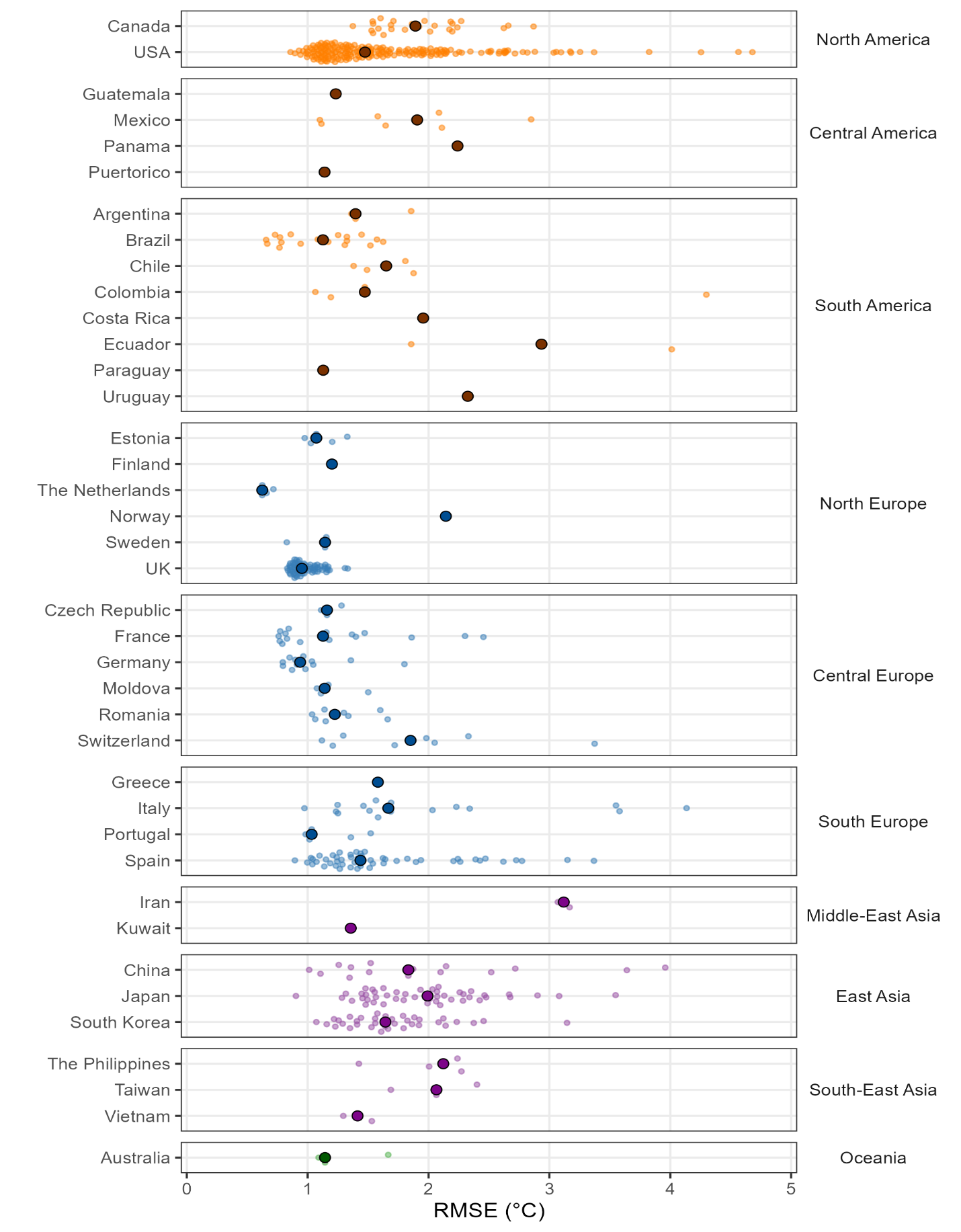


**Figure S4: Root Mean Square Error (RMSE) computed as the squared difference between MCC weather station and ERA5-Land daily mean temperature (°C) across the 612 locations used in the study grouped by 39 countries and 10 regions. The circle in each country panel depicts the median RMSE.**


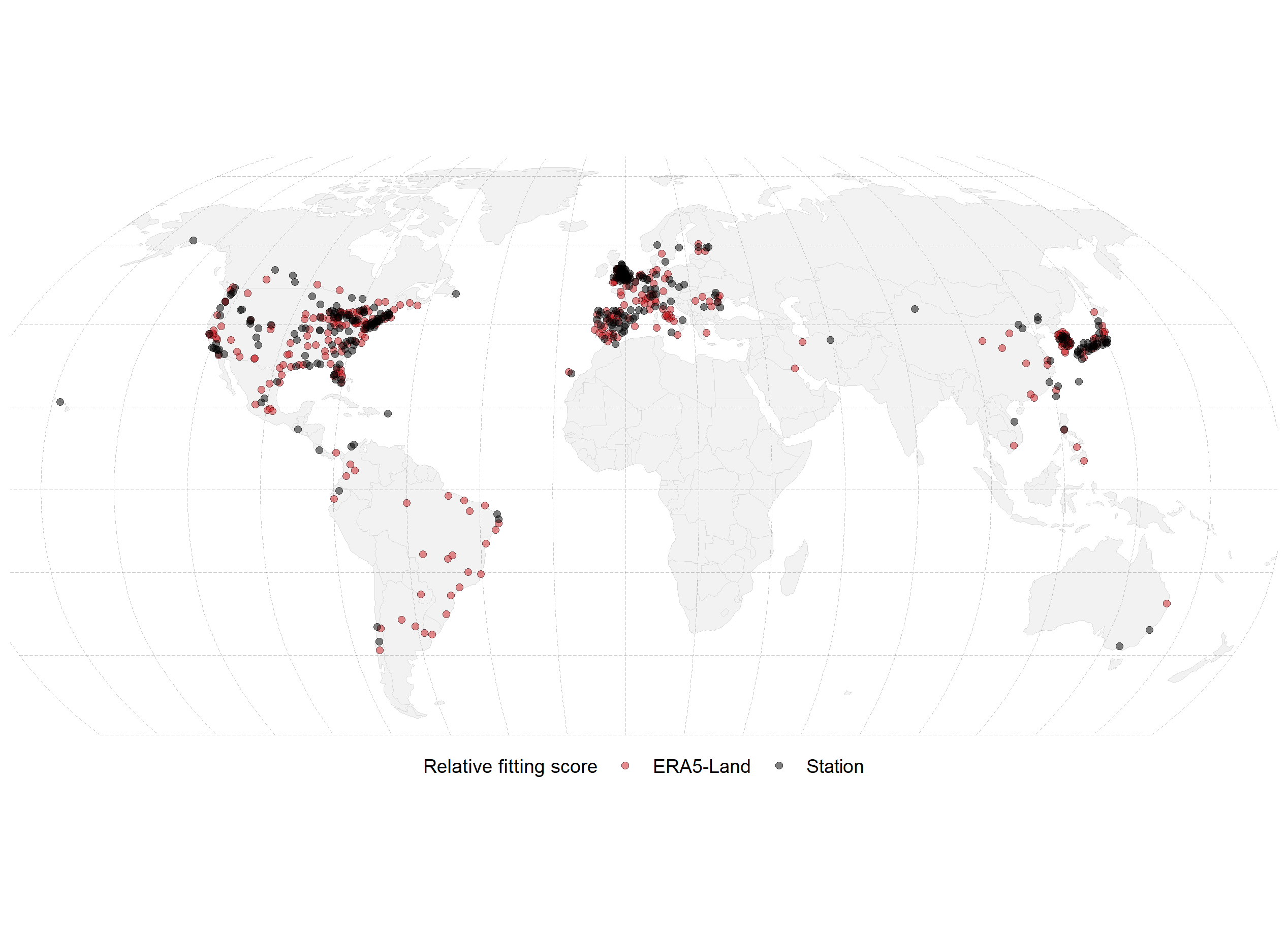


**Figure S5:** Relative Fitting Score (RFS) for station observations and ERA5-Land. A negative RFS represented by red dots implies a superior predictive ability of ERA5-Land at the location. Conversely a positive RFS represented by black dots indicates a better performance of ground station observations at the location.

**Sensitivity results using ERA5**


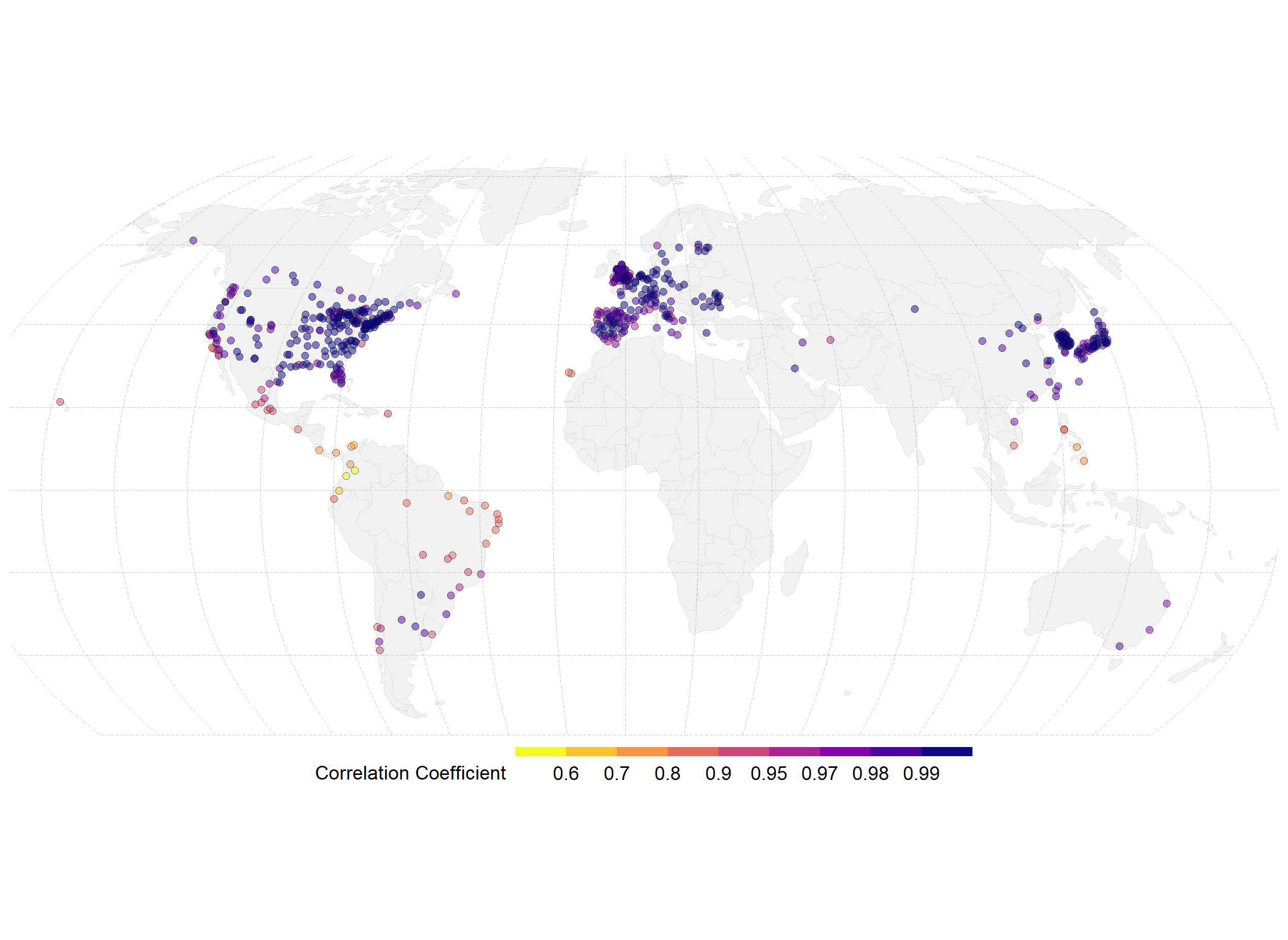


**Figure S6: Correlation between MCC weather station and ERA5 daily mean temperature (°C) across the 612 locations used in the study.**


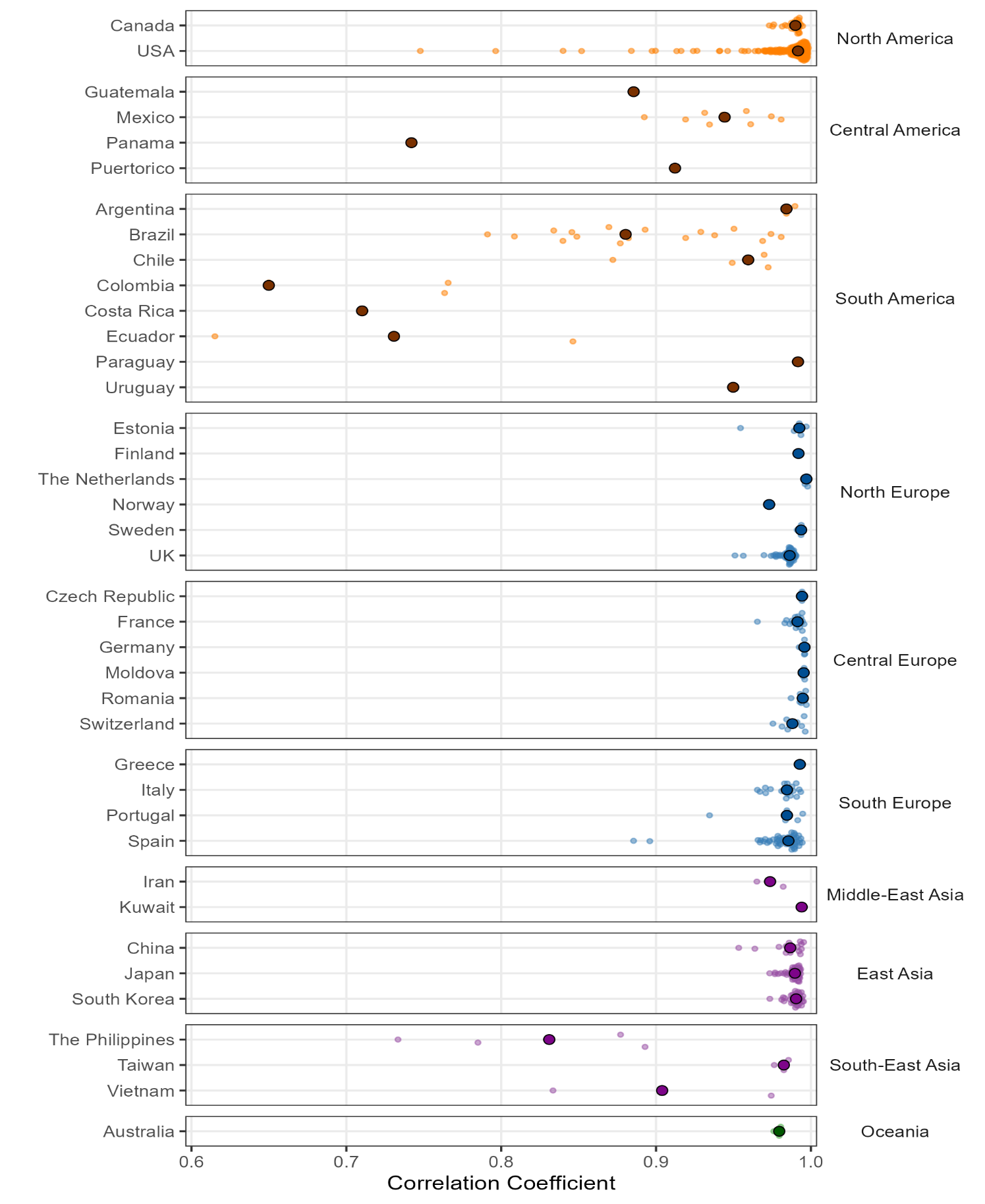


**Figure S7: Correlation between MCC weather station and ERA5 daily mean temperature (°C) across the 612 locations used in the study grouped by 39 countries and 10 regions. The circle in each country panel depicts the median correlation.**


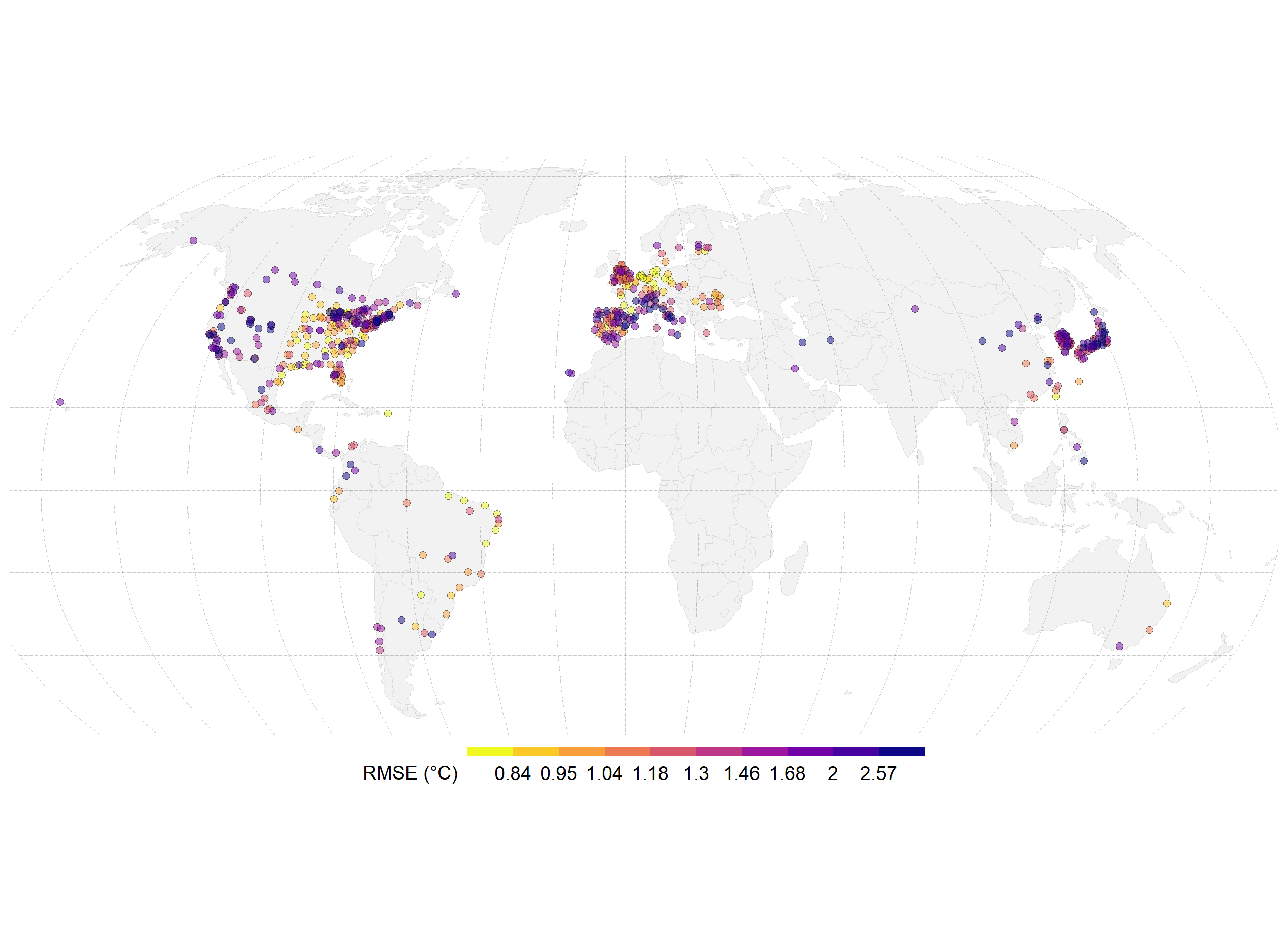


**Figure S8: Root Mean Square Error (RMSE) computed as the squared difference between MCC weather station and ERA5 daily mean temperature (°C) across the 612 locations used in the study.**


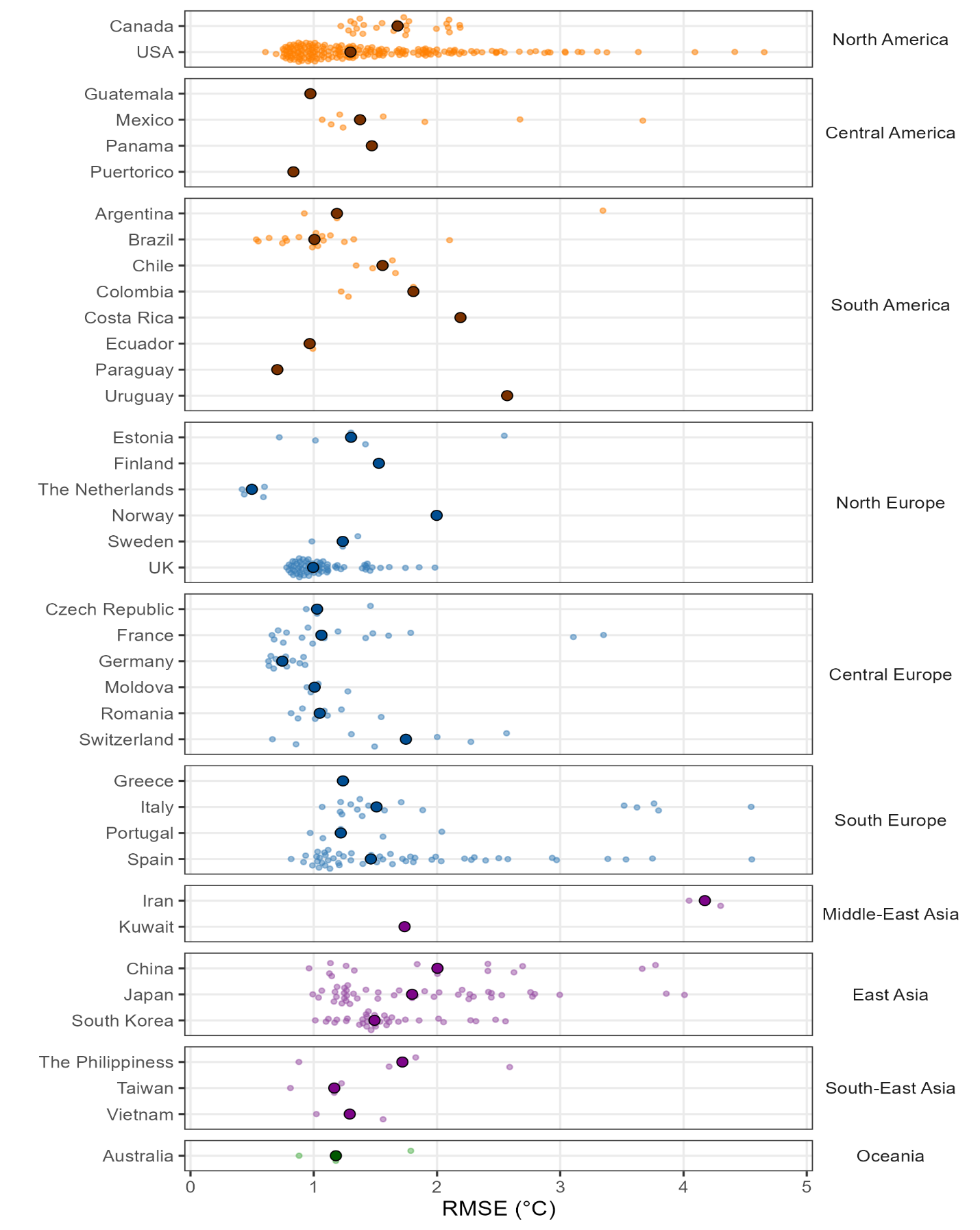


**Figure S9: Root Mean Square Error (RMSE) computed as the squared difference between MCC weather station and ERA5 daily mean temperature (°C) across the 612 locations used in the study grouped by 39 countries and 10 regions. The circle in each country panel depicts the median RMSE.**


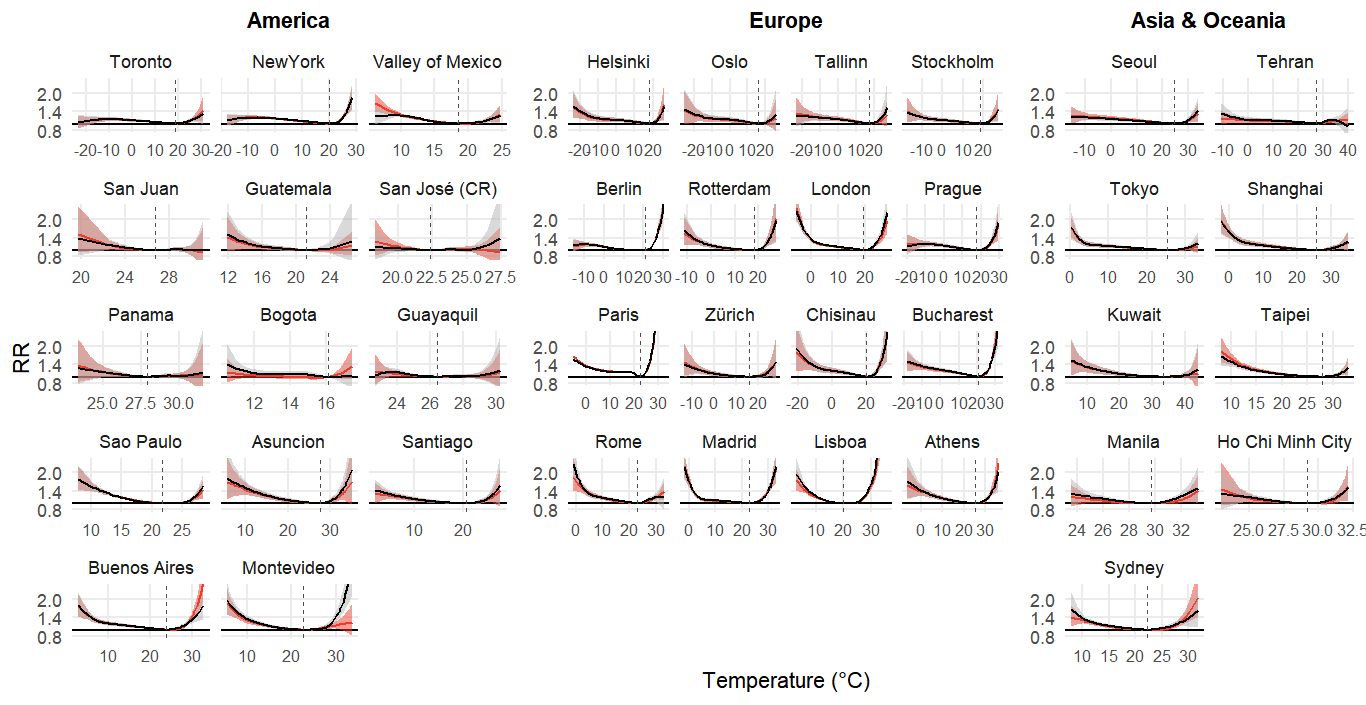


**Figure S10:** Overall cumulative exposure-response associations in selective cities representative of the 39 countries (station observations **-black** and ERA5 -red, with 95% confidence intervals (CI) -shaded, see Methods). Exposure-response associations as best linear unbiased predicition (BLUP) using the distribution drawn from station temperature. Dashed vertical grey lines are the minimum mortality temperatures (MMTs). RR=relative risk.


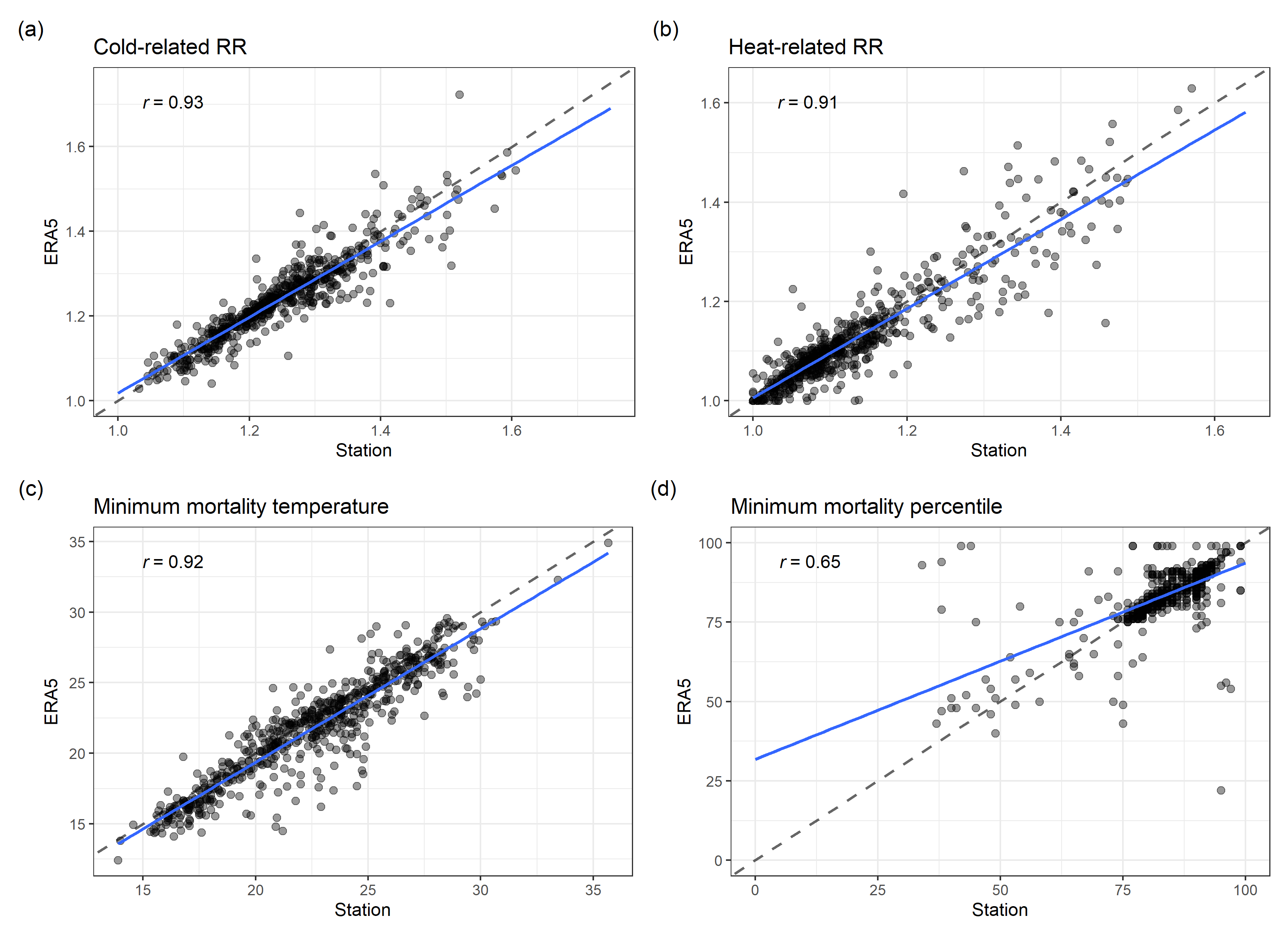


**Figure S11:** Scatterplots of: (a) and (b) cumulative relative risks (RRs) at the 1^st^ and the 99th percentile respectively; (c) Minimum mortality temperature (MMT) and (d) Minimum mortality percentile (MMP). The RRs, MMP and MMT are based on the station and ERA5 temperatures of the best linear unbiased predictions (BLUPs) for individual cities. Blue lines and the r values represent the linear regression trend and the correlation coefficient of compared variables respectively. The dashed black line represents the 1:1 line.


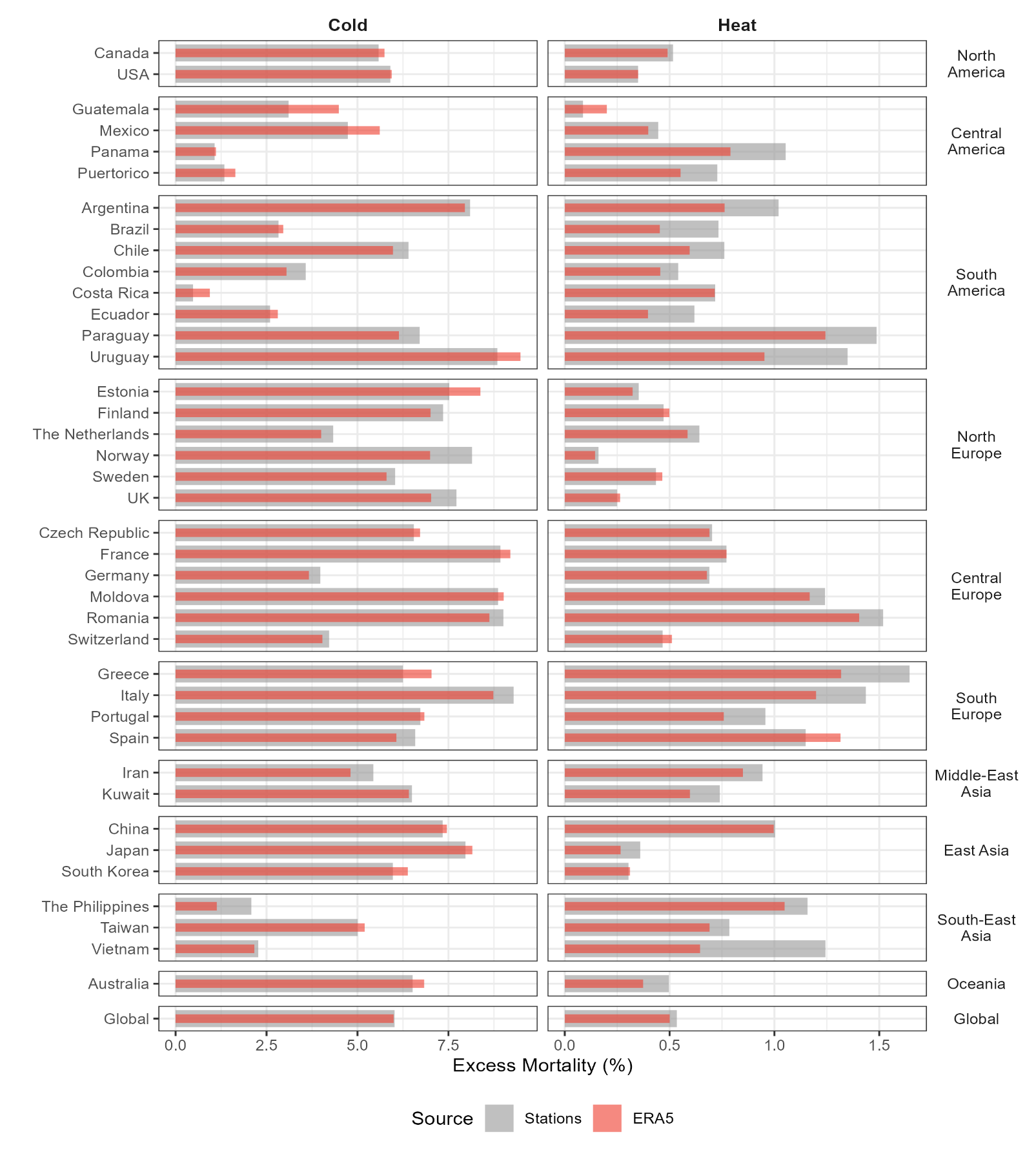


**Figure S12**: Fraction of all-cause excess mortality (%) due to cold and hot temperatures by countries and all 612 locations (Global) estimated using station observations (gray) and ERA5 (red). The bar plots represent the excess deaths. The 95% empirical confidence intervals (eCI) computed using Monte Carlo simulations (see Methods) are reported in Table S3. Note: the range of y-axes are different in the two panels


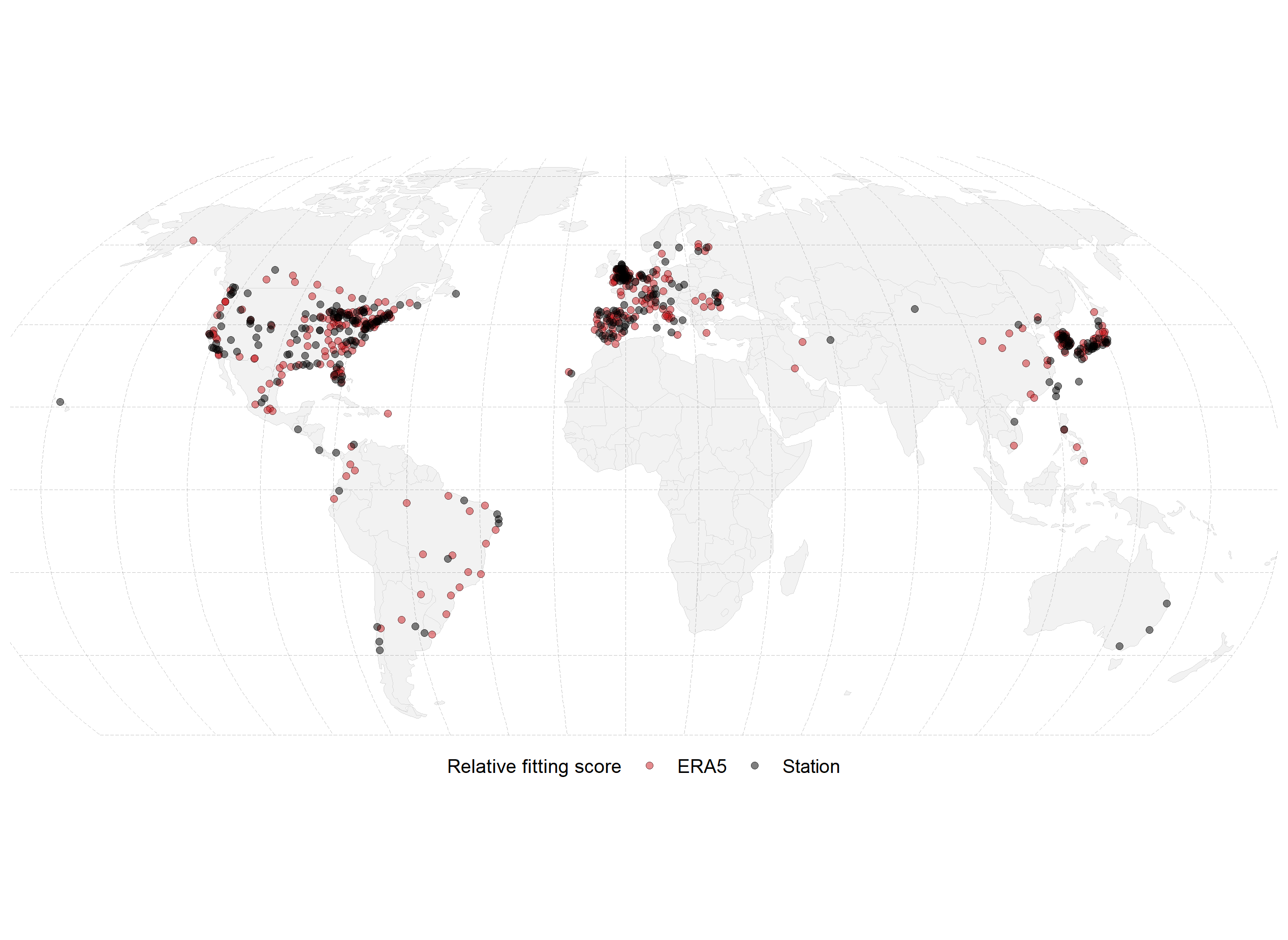


**Figure S13:** Relative fitting score (RFS) for station observations and ERA5. A negative RFS represented by red dots implies a superior predictive ability of ERA5 at the location. Conversely a positive RFS represented by black dots indicates a better performance of ground station observations at the location.


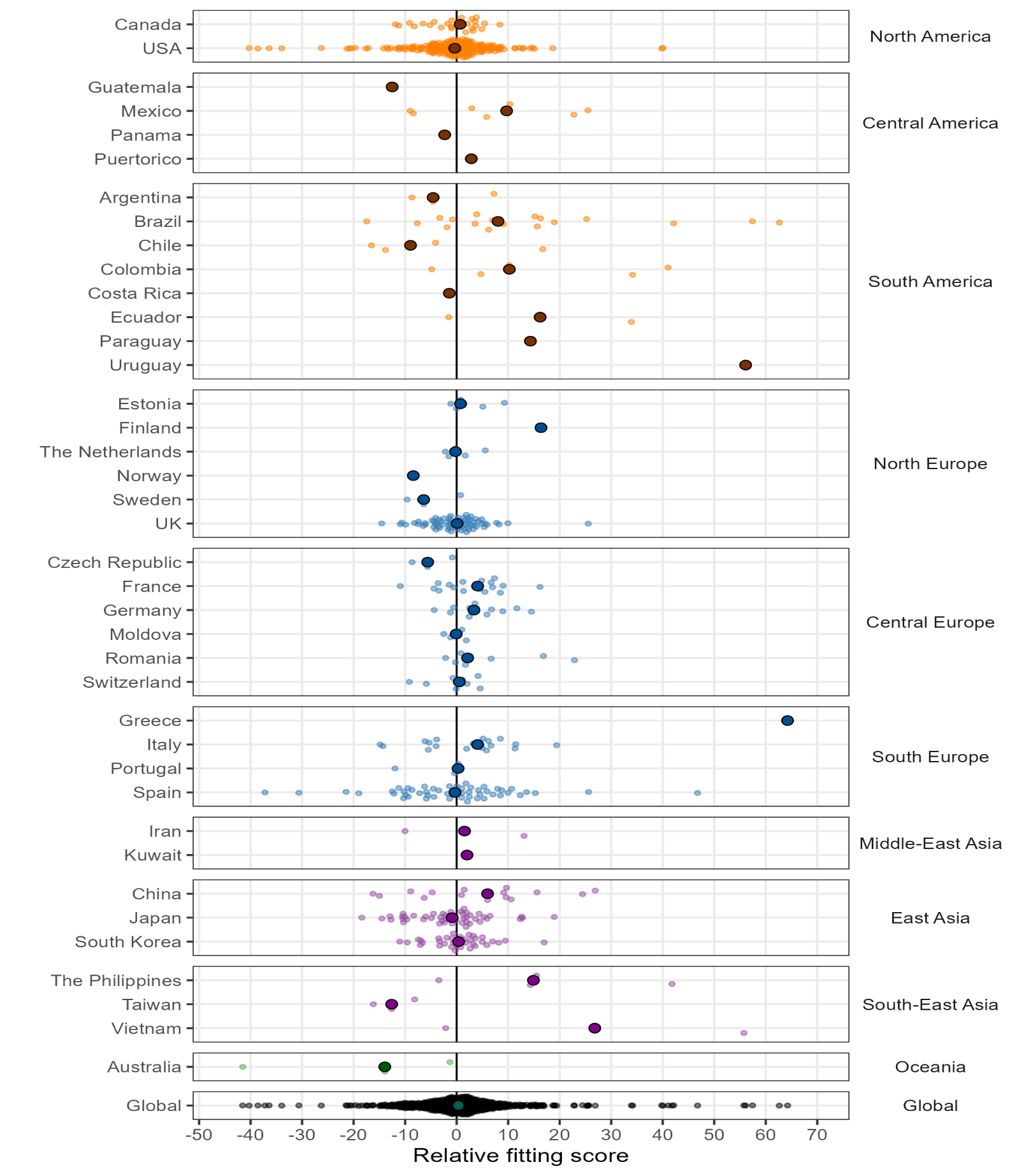


**Figure S14:** Relative fitting score (RFS) for station observations and ERA5 by country. A negative score indicates a better performance of the model based on ERA5 temperature relative to the model fitted using station temperature at a location. The shaded circle in each country panel depicts the median RFS. ‘Global’ implies all 612 locations used in the study.

**Table S1** Description of the observed temperature (˚C) and mortality data for the MCC locations used in the study. Where not indicated otherwise, the mean daily temperature is computed as the 24-hour average based on hourly measurements from weather station.

| **Country** | **No. and Type of Locations** | **Time Period** | **Mortality Data (Type – Source)** | **Meteorological Data (Source and Remarks)** |
| --- | --- | --- | --- | --- |
| Argentina | 3 cities | 2005-2015 | Non-external causes only (ICD-9: 0-799; ICD-10: A00-R99) - National Ministry of Health (Ministerio de Salud de la Nación) | Weather station located within each city - Servicio Meteorológico Nacional (National Weather Service) |
| Australia | 3 cities | 1988-2009 | Non-external causes only (ICD-9: 0-799; ICD-10: A00-R99)  - Australian Bureau of Statistics | Weather stations located within ≤30 km  of each city – Australian Bureau of Meteorology |
| Brazil | 18 cities | 1997-2011 | Non-external causes only (ICD-9: 0-799; ICD-10: A00-R99)  - Ministry of Health | Weather stations located within the urban area - National Institute of Meteorology of Brazil |
| Canada | 26 (25 census metropolitan areas -CMA- and 1 city) | 1986-2015 | All causes - Canadian Mortality Database | Nearest weather station - Environment Canada |
| Chile | 4 cities | 2004-2014 | All causes - Department of Statistics and Information, Ministry of Health (Departamento de  Estadísticas e Información de Salud, Ministerio de Salud) | Nearest weather station - Ministerio del Medio Ambiente, Sistema de Información Nacional de Calidad del Aire (SINCA) |
| China | 15 cities | 1996-2015 | Non-external causes only (ICD-9: 0-799; ICD-10: A00-R99)  - Municipal Center for Disease Control and Prevention in each city | Weather station located within each city - China Meteorological Data Sharing Service  System (http://data.cma.cn/) |
| Colombia | 5 cities | 1998-2013 | All causes - National Administrative Department of Statistics (DANE) | Nearest weather station - Instituto de Hidrología, Meteorología y Estudios Ambientales de Colombia (IDEAM) |
| Costa Rica | 1 city | 2000-2017 | All causes - Instituto Nacional  de Estadística y Censo. Open Access. | Meteorological data - World Meteorological Orgnaization – National Oceanic and Atmospheric Administration (WMO-NOAA) Surface Data Hourly Global (DS3505) |
| Czech Republic | 3 cities | 1994-2015 | All causes - Czech Statistical Office and the Institute of Health Information and Statistics | Weather station located within each city - Czech Hydrometeorological Institute (measurements in standard climatic terms 7:00, 14:00 and 21:00 local time, and daily means) |
| Ecuador | 2 cities | 2014-2018 | All causes - Estonian Causes of Death Registry | Meteorological data - WMO-NOAA Surface Data Hourly Global (DS3505) |
| Estonia | 5 cities | 1997-2018 | All causes - Estonian Causes of Death Registry | Nearest weather station - Estonian Environment Agency |
| Finland | 1 city | 1994-2014 | All causes - Statistics Finland | Mean daily temperature - Finnish Meteorological Institute. The weather stations around the country were interpolated onto a 10×10 km grid covering the whole of Finland, using a Kriging model |
| France | 18 cities | 2000-2014 | All causes - French National Institute of Health and Medical Research (CepiDC) | Nearest weather station, usually the airport -Meteo France |
| Germany | 12 cities | 1993-2015 | All causes - Research Data Centres of the Federation and the Federal States of Germany (Forschungsdatenzentrum der Statistischen Ämter des Bundes und der Länder) | Nearest weather station - Climate Data Centre of the German National Meteorological Service (Deutscher Wetterdienst) |
| Greece | 1 city | 2001-2010 | All causes - Hellenic Statistical Authority | National observatory of Athens (<http://www.noa.gr/>) from site “Thisio” located in the city of Athens. |
| Guatemala | 1 city | 2009-2016 | All causes - Instituto Nacional de Estadística, Unidad de Estadística de Salud. | Nearest weather station - Instituto Nacional de Sismología, Vulancología, Meteorología y Hidrología. |
| Iran | 2 cities | 2002-2015 | All causes - Ferdows organization of Mashhad Municipality | Nearest weather station - IRAN Meteorological  Organization (IRIMO) (http://www.irimo.ir) |
| Italy | 18 cities | 2006-2015 | All causes - local mortality registries and the rapid mortality surveillance system | Airport monitoring station located closest to the city centre - Meteorological Service of the Italian Air Force. 24-h average based on 6-h measurements |
| Japan | 47 cities | 2011-2015 | All causes - Ministry of Health, Labour and Welfare | Weather station located within the urban area of the capital city - Japan Meteorology Agency (JMA) |
| Kuwait | 1 city | 2000-2016 | Non-external causes only (ICD-9: 0-799; ICD-10: A00-R99) - National Center for Health Information, Ministry of Health | Nearest weather station – the Directorate General of Civil Aviation (Kuwait Airport) and Kuwait's Environmental Public Authority. |
| Mexico | 10 cities | 1998-2014 | All causes - National Institute of Statistics, Geography and Informatics | Weather station located within the urban area or at a near airport - Primarily Servicio Meteorológico Nacional (SMN) Estaciones Sinópticas Meteorológicas (ESIMES), Estaciones Meteorológicas Automáticas (EMAS)* and Observatories. Otherwise (i) Instituto Nacional de Ecología y Cambio Climático (INECC). (ii) Red de Meteorología y Radiación Solar (REDMET) from the Sistema de Monitoreo Atmosférico de la Ciudad de México (SIMAT). (iii) WMO stations from the Weather Underground.) |
| Moldova | 4 cities | 2001-2010 | All causes - National Centre for Health Management | Nearest weather station - State Hydrometeorological Service, Moldova. Mean daily temperature computed as the average between daily minimum and maximum |
| The Netherlands | 5 cities | 1995-2016 | All causes - Statistics Netherlands | Nearest weather station – Royal Dutch Meteorological Institute (KNMI) |
| Norway | 1 city | 1985-2018 | All causes - Norwegian Cause of Death registry | Mean daily temperature based on the observational modelled dataset from the Norwegian Meteorological Institute. |
| Panama | 1 city | 2013-2016 | All causes Instituto Nacional de Estadística y Censo, Centro de Información Estadística. | Open access temperature data - Empresa de Transmisión Eléctrica, S.A. (ETESA) |
| Paraguay | 1 city | 2004-2019 | All causes - Ministerio de Salud Pública y Bienestar Social, Dirección General de Información Estratégica en Salud,  Subsistema de Información de Estadísticas Vitales | Meteorological data - WMO-NOAA Surface Data Hourly Global (DS3505) |
| The Philippines | 4 cities | 2006-2010 | All causes - Philippine Statistics Agency | Station in or near location - Philippine Atmospheric Geophysical and Astronomical Services Administration |
| Portugal | 5 cities | 1985-2018 | All causes - Statistics Portugal | Meteorological data - WMO-NOAA Surface Data Hourly Global (DS3505) |
| Puerto Rico | 1 city | 2009-2016 | All causes - Instituto de Estadísticas  Vitales de Puerto Rico, Área de Estadísticas  Vitales del Departamento de Salud | Meteorological data - WMO-NOAA Surface Data Hourly Global (DS3505) |
| Romania | 8 cities | 1994-2016 | All causes - Romanian National Institute of Statistics | Mean daily temperature - National Meteorological Administration of Romania (NMARO, accessed from https://www.ecad.eu/) |
| South Korea | 36 cities | 1997-2018 | All causes – South Korea Bureau of Statistics | Weather station located within the location - Korea Meteorological Administration |
| Spain | 52 cities | 1990-2014 | Non–external causes (ICD–9: 0–799; ICD–10: A00–R99) - Spain National Institute of Statistics. | Weather station in the location or at a near airport - Spain National Meteorology Agency |
| Sweden | 3 cities | 1990-2016 | All causes - Swedish Cause of Death Register at the Swedish National Board of Health and Welfare | Weather station in the location or at a near airport (Environment and Health Administration) |
| Switzerland | 8 (7 cities and 1 metropolitan  area -Lugano) | 1995-2013 | Non–external causes only other than accidents (ICD–10codes A00–R99, V01–V99, W00–X59) - Federal Office of Statistics | Weather station located within or near the urban area for each city - IDAWEB database (a service provided by MeteoSwiss, the Swiss Federal Office of Meteorology and Climatology). |
| Taiwan | 3 cities | 1994-2014 | All causes - Department of Health | 1-15 stations per location - Taiwan Environmental Protection Administration |
| UK | 70 cities | 1990-2016 | All causes - Office of National Statistics. | 29 stations on average per location - British Atmospheric Data Centre (BADC) |
| Uruguay | 1 city | 2012-2016 | Non-external causes - Ministerio de Salud Publica (MSP) | Nearest weather station - Instituto Uruguayo de Meteorología (INUMET) |
| USA | 211 cities | 1985-2006 | All causes - National Center for Health Statistics | Weather station closest to the city centre -National Climatic Data Center (NCDC) of NOAA |
| Vietnam | 2 cities | 2009-2013 | All causes - Provincial Department of Health | Weather station at city airport |

**Table S2:** Detailed summary statistics

File **Supplementary_Table_S2.xlsx** goes here.

**Table S3:** Fraction of all-cause excess mortality (%) due to cold and heat by countries and all 612 locations (Global) estimated using station observations, ERA5-Land and ERA5. The 95% empirical confidence intervals (eCI) computed using Monte Carlo simulations (see Methods) are reported in box brackets.

| **Country** | **Estimated excess mortality due to cold [eCI]** | | | **Estimated excess mortality due to heat [eCI]** | | |
| --- | --- | --- | --- | --- | --- | --- |
|  | **Station Observations** | **ERA5-Land** | **ERA5** | **Station Observations** | **ERA5-Land** | **ERA5** |
| Argentina | 8.10 [6.33 - 9.90] | 8.07 [6.18 - 9.87] | 7.95 [6.25 - 9.59] | 1.02 [0.65 - 1.35] | 0.62 [0.43 - 0.81] | 0.76 [0.49 - 1.02] |
| Australia | 6.52 [4.53 - 8.43] | 7.42 [5.23 - 9.36] | 6.83 [4.79 - 8.87] | 0.50 [0.25 - 0.71] | 0.39 [0.27 - 0.48] | 0.37 [0.26 - 0.47] |
| Brazil | 2.83 [2.29 - 3.38] | 2.90 [2.07 - 3.69] | 2.96 [2.11 - 3.80] | 0.73 [0.47 - 0.99] | 0.70 [-0.14 - 1.50] | 0.45 [0.23 - 0.62] |
| Canada | 5.58 [4.71 - 6.42] | 6.53 [5.65 - 7.39] | 5.74 [4.79 - 6.63] | 0.52 [0.38 - 0.64] | 0.43 [0.31 - 0.55] | 0.49 [0.37 - 0.60] |
| Chile | 6.40 [3.61 - 9.12] | 5.51 [3.02 - 7.97] | 5.98 [3.36 - 8.48] | 0.76 [0.24 - 1.27] | 0.48 [-0.09 - 0.99] | 0.60 [0.08 - 1.06] |
| China | 7.34 [6.06 - 8.57] | 7.35 [6.29 - 8.49] | 7.46 [6.22 - 8.59] | 1.00 [0.73 - 1.23] | 0.88 [0.63 - 1.10] | 1.00 [0.74 - 1.25] |
| Colombia | 3.58 [-0.51 - 7.30] | 3.24 [1.27 - 5.12] | 3.05 [0.96 - 5.13] | 0.54 [0.06 - 1.00] | 0.50 [0.19 - 0.78] | 0.46 [0.20 - 0.74] |
| Costa Rica | 0.48 [-0.80 - 1.67] | 1.26 [-0.81 - 3.11] | 0.94 [-0.53 - 2.42] | 0.72 [-1.38 - 2.68] | 0.81 [-0.74 - 2.24] | 0.72 [-0.76 - 2.31] |
| Czech Republic | 6.55 [4.59 - 8.50] | 6.85 [4.97 - 8.69] | 6.72 [4.75 - 8.53] | 0.70 [0.38 - 0.98] | 0.62 [0.35 - 0.86] | 0.69 [0.42 - 0.93] |
| Ecuador | 2.60 [0.53 - 4.62] | 4.47 [-2.40 - 11.18] | 2.81 [0.50 - 5.05] | 0.62 [-0.26 - 1.43] | 0.03 [-0.15 - 0.17] | 0.40 [-0.38 - 1.17] |
| Estonia | 7.52 [5.05 - 9.85] | 8.36 [6.34 - 10.41] | 8.38 [5.37 - 11.07] | 0.35 [0.14 - 0.54] | 0.36 [0.07 - 0.61] | 0.32 [0.09 - 0.53] |
| Finland | 7.35 [3.09 - 11.39] | 7.56 [3.76 - 10.93] | 7.01 [2.41 - 10.78] | 0.47 [0.13 - 0.76] | 0.42 [0.08 - 0.73] | 0.50 [0.21 - 0.78] |
| France | 8.93 [7.78 - 9.94] | 9.28 [8.06 - 10.44] | 9.20 [7.98 - 10.40] | 0.77 [0.70 - 0.83] | 0.70 [0.63 - 0.77] | 0.77 [0.72 - 0.82] |
| Germany | 3.98 [3.01 - 4.80] | 3.90 [2.95 - 4.71] | 3.66 [2.75 - 4.55] | 0.69 [0.60 - 0.76] | 0.61 [0.54 - 0.67] | 0.68 [0.59 - 0.74] |
| Greece | 6.25 [3.22 - 9.49] | 7.20 [4.20 - 10.20] | 7.03 [4.14 - 9.81] | 1.64 [0.99 - 2.34] | 1.14 [0.44 - 1.81] | 1.32 [0.67 - 2.00] |
| Guatemala | 3.11 [-0.50 - 6.63] | 3.77 [-1.21 - 8.38] | 4.49 [-0.94 - 9.60] | 0.09 [-0.29 - 0.43] | 0.29 [-0.13 - 0.65] | 0.20 [-0.31 - 0.64] |
| Iran | 5.44 [2.52 - 8.21] | 5.00 [2.05 - 7.76] | 4.81 [1.96 - 7.34] | 0.94 [0.50 - 1.43] | 0.88 [0.31 - 1.37] | 0.85 [0.31 - 1.32] |
| Italy | 9.29 [8.06 - 10.42] | 9.22 [7.71 - 10.63] | 8.73 [7.32 - 10.07] | 1.44 [1.19 - 1.64] | 1.24 [1.02 - 1.45] | 1.20 [0.96 - 1.41] |
| Japan | 7.97 [6.93 - 8.96] | 8.49 [7.50 - 9.46] | 8.16 [7.12 - 9.08] | 0.36 [0.23 - 0.47] | 0.25 [0.13 - 0.36] | 0.27 [0.15 - 0.36] |
| Kuwait | 6.50 [2.60 - 9.91] | 6.02 [2.01 - 9.47] | 6.41 [2.59 - 10.19] | 0.74 [-0.35 - 1.70] | 0.69 [-0.37 - 1.71] | 0.60 [-0.47 - 1.61] |
| Mexico | 4.73 [3.57 - 5.84] | 5.38 [4.30 - 6.30] | 5.61 [4.49 - 6.58] | 0.45 [0.22 - 0.68] | 0.44 [0.26 - 0.61] | 0.40 [0.24 - 0.53] |
| Moldova | 8.86 [5.08 - 12.44] | 9.11 [5.45 - 12.46] | 9.02 [5.41 - 12.00] | 1.24 [0.41 - 1.95] | 1.07 [0.45 - 1.64] | 1.17 [0.46 - 1.80] |
| The Netherlands | 4.33 [2.73 - 5.86] | 4.07 [2.49 - 5.55] | 4.00 [2.20 - 5.50] | 0.64 [0.44 - 0.82] | 0.52 [0.35 - 0.68] | 0.59 [0.41 - 0.75] |
| Norway | 8.15 [3.70 - 12.49] | 8.17 [2.85 - 13.02] | 7.00 [1.39 - 11.84] | 0.16 [-0.15 - 0.44] | 0.17 [-0.15 - 0.47] | 0.14 [-0.19 - 0.47] |
| Panama | 1.07 [-0.33 - 2.46] | 1.30 [-0.56 - 3.10] | 1.11 [-0.71 - 3.00] | 1.05 [-1.31 - 3.29] | 0.94 [-0.92 - 2.75] | 0.79 [-1.22 - 2.56] |
| Paraguay | 6.71 [3.31 - 9.92] | 5.19 [2.27 - 7.80] | 6.14 [3.21 - 9.13] | 1.49 [0.66 - 2.31] | 0.95 [0.10 - 1.71] | 1.24 [0.47 - 1.99] |
| The Philippines | 2.08 [0.88 - 3.26] | 1.14 [0.43 - 1.86] | 1.13 [-1.13 - 3.24] | 1.16 [0.65 - 1.64] | 1.00 [0.46 - 1.49] | 1.05 [0.42 - 1.65] |
| Portugal | 6.73 [5.65 - 7.84] | 7.18 [5.59 - 8.80] | 6.84 [5.33 - 8.17] | 0.96 [0.74 - 1.15] | 0.81 [0.71 - 0.91] | 0.76 [0.62 - 0.89] |
| Puerto Rico | 1.34 [-0.32 - 2.88] | 2.14 [-0.42 - 4.32] | 1.64 [-0.68 - 3.74] | 0.73 [-1.40 - 2.91] | 0.71 [-0.87 - 2.27] | 0.55 [-1.17 - 2.16] |
| Romania | 9.01 [7.43 - 10.48] | 8.97 [7.41 - 10.43] | 8.62 [7.12 - 9.98] | 1.52 [1.24 - 1.76] | 1.21 [0.96 - 1.45] | 1.40 [1.13 - 1.64] |
| South Korea | 5.97 [4.86 - 7.15] | 6.69 [5.34 - 7.86] | 6.38 [5.28 - 7.60] | 0.30 [0.17 - 0.44] | 0.31 [0.18 - 0.43] | 0.31 [0.16 - 0.43] |
| Spain | 6.59 [5.86 - 7.28] | 6.37 [5.69 - 7.00] | 6.07 [5.41 - 6.69] | 1.15 [1.03 - 1.28] | 1.20 [1.06 - 1.33] | 1.32 [1.18 - 1.43] |
| Sweden | 6.03 [3.64 - 8.37] | 6.58 [4.41 - 8.82] | 5.80 [3.62 - 7.83] | 0.43 [0.22 - 0.64] | 0.44 [0.23 - 0.65] | 0.46 [0.26 - 0.67] |
| Switzerland | 4.22 [2.36 - 5.93] | 4.22 [2.59 - 5.85] | 4.03 [2.38 - 5.35] | 0.47 [0.17 - 0.72] | 0.44 [0.20 - 0.71] | 0.51 [0.23 - 0.76] |
| Taiwan | 5.00 [3.60 - 6.36] | 5.38 [4.10 - 6.61] | 5.20 [3.85 - 6.61] | 0.79 [0.31 - 1.24] | 0.64 [0.22 - 1.07] | 0.69 [0.30 - 1.08] |
| UK | 7.72 [7.06 - 8.33] | 7.17 [6.58 - 7.73] | 7.02 [6.35 - 7.70] | 0.25 [0.20 - 0.29] | 0.26 [0.22 - 0.30] | 0.26 [0.22 - 0.30] |
| Uruguay | 8.85 [5.66 - 11.56] | 9.22 [6.01 - 12.21] | 9.48 [6.46 - 12.80] | 1.35 [0.57 - 2.02] | 1.01 [0.33 - 1.74] | 0.95 [0.22 - 1.65] |
| USA | 5.90 [5.60 - 6.18] | 6.25 [5.98 - 6.51] | 5.94 [5.59 - 6.21] | 0.35 [0.30 - 0.39] | 0.33 [0.28 - 0.38] | 0.35 [0.29 - 0.40] |
| Vietnam | 2.27 [0.00 - 4.55] | 1.83 [0.25 - 3.23] | 2.16 [-0.88 - 5.18] | 1.24 [0.33 - 2.02] | 1.04 [-0.07 - 1.94] | 0.65 [-0.15 - 1.35] |
| Global | 6.02 [5.80 - 6.18] | 6.25 [6.05 - 6.41] | 5.99 [5.79 - 6.16] | 0.53 [0.50 - 0.56] | 0.49 [0.43 - 0.53] | 0.50 [0.47 - 0.52] |

**Table S4:** Summary of the Relative Fitting Score (RFS) by countries.

| **Country** | **Total number of locations in the study** | **Number of locations where ERA5-Land (Station Observations) perform better** |
| --- | --- | --- |
| Argentina | 3 | 0 (3) |
| Australia | 3 | 2 (1) |
| Brazil | 18 | 2 (16) |
| Canada | 26 | 12 (14) |
| Chile | 4 | 2 (2) |
| China | 15 | 7 (8) |
| Colombia | 5 | 2 (3) |
| Costa Rica | 1 | 1 (0) |
| Czech Republic | 3 | 3 (0) |
| Ecuador | 2 | 1 (1) |
| Estonia | 5 | 3 (2) |
| Finland | 1 | 0 (1) |
| France | 18 | 7 (11) |
| Germany | 12 | 5 (7) |
| Greece | 1 | 0 (1) |
| Guatemala | 1 | 1 (0) |
| Iran | 2 | 1 (1) |
| Italy | 18 | 5 (13) |
| Japan | 47 | 31 (16) |
| Kuwait | 1 | 0 (1) |
| Mexico | 10 | 2 (8) |
| Moldova | 4 | 2 (2) |
| The Netherlands | 5 | 3 (2) |
| Norway | 1 | 1 (0) |
| Panama | 1 | 0 (1) |
| Paraguay | 1 | 0 (1) |
| The Philippines | 4 | 1 (3) |
| Portugal | 5 | 2 (3) |
| Puerto Rico | 1 | 1 (0) |
| Romania | 8 | 3 (5) |
| South Korea | 36 | 10 (26) |
| Spain | 52 | 29 (23) |
| Sweden | 3 | 2 (1) |
| Switzerland | 8 | 3 (5) |
| Taiwan | 3 | 2 (1) |
| UK | 70 | 39 (31) |
| Uruguay | 1 | 0 (1) |
| USA | 211 | 106 (105) |
| Vietnam | 2 | 1 (1) |
| Global | 612 | 292 (320) |

**MCC Collaborative Research Network.**

Barrak Alahmad, Rosana Abrutzky, Paulo Hilario Nascimento Saldiva, Patricia Matus Correa, Nicolás Valdés Orteg, Haidong Kan, Samuel Osorio, Ene Indermitte, Jouni J. K. Jaakkola, Niilo Ryti, Alexandra Schneider, Veronika Huber, Klea Katsouyanni, Antonis Analitis, Alireza Entezari, Fatemeh Mayvaneh, Paola Michelozzi, Francesca de'Donato, Masahiro Hashizume, Yoonhee Kim, Magali Hurtado Diaz, César De la Cruz Valencia, Ala Overcenco, Danny Houthuijs, Caroline Ameling, Shilpa Rao, Xerxes Seposo, Baltazar Nunes, Iulian-Horia Holobaca, Ho Kim, Whanhee Lee, Carmen Íñiguez, Bertil Forsberg, Christofer Åström, Martina S. Ragettli, Yue-Liang Leon Guo, Bing-Yu Chen, Valentina Colistro, Antonella Zanobetti, Joel Schwartz, Tran Ngoc Dang and Do Van Dung.
